# PA3225 Is a Transcriptional Repressor of Antibiotic Resistance Mechanisms in Pseudomonas aeruginosa

**DOI:** 10.1128/AAC.02114-16

**Published:** 2017-07-25

**Authors:** Clayton W. Hall, Li Zhang, Thien-Fah Mah

**Affiliations:** Department of Biochemistry, Microbiology, and Immunology, University of Ottawa, Ottawa, Ontario, Canada

**Keywords:** Pseudomonas aeruginosa, antibiotic resistance, multidrug efflux pump, transcriptional regulator

## Abstract

The *tssABC1* locus is part of the Hcp secretion island I (HSI-I) type VI secretion system (T6SS) in Pseudomonas aeruginosa. Previous work implicated the *tssC1* gene in P. aeruginosa biofilm-specific antibiotic resistance, and *tssC1* is preferentially expressed in biofilms compared to planktonic cells. Using a DNA-dependent protein pulldown approach, we discovered that PA3225, an uncharacterized LysR-type transcriptional regulator, specifically bound to the *tssABC1* upstream regulatory region. The deletion of PA3225 led to a 2-fold decrease in *tssA1* expression levels in planktonic cells compared to the wild type, and *tssA1* expression was slightly reduced in ΔPA3225 biofilms compared to wild-type biofilms. Intriguingly, further investigations revealed that the ΔPA3225 mutant was less susceptible to multiple, structurally unrelated antibiotics with various mechanisms of action when grown planktonically. The ΔPA3225 mutant was additionally more resistant to ciprofloxacin when grown in a biofilm. The decreased antibiotic susceptibility of the ΔPA3225 strain was linked to the transcriptional upregulation of the MexAB-OprM efflux pump. By using transcriptome sequencing (RNA-seq), other PA3225-regulated genes were identified, and the products of these genes, such as the putative ABC transporter PA3228, may also contribute to antibiotic resistance.

## INTRODUCTION

In Gram-negative bacteria, type VI secretion systems (T6SSs) represent a novel class of secretion systems that are structurally and mechanistically similar to components of the T4 phage (reviewed in reference 1). The opportunistic pathogen Pseudomonas aeruginosa possesses three characterized T6SSs, known as Hcp secretion island I (HSI-I), HSI-II, and HSI-III (2). The HSI-I T6SS secretes several toxins and is involved in interbacterial competition (reviewed in reference 3). The HSI-T6SS locus is made up of several components, including the gene products of the *tssABC1* operon (2). Intriguingly, via an unknown mechanism, the *tssC1* gene is required for biofilm-specific antibiotic resistance in P. aeruginosa: a Δ*tssC1* mutant is 4-fold more susceptible to tobramycin than the wild-type strain only when grown in a biofilm (4). The biofilm-specific antibiotic resistance phenotype of the Δ*tssC1* mutant is explained by the fact that *tssC1* is more highly expressed in wild-type biofilms than in wild-type planktonic cells (4).

The *tssABC1* locus is posttranscriptionally regulated by the actions of RetS (2), an orphan sensor kinase that mediates the switch between acute and chronic infections in P. aeruginosa (5, 6). RetS forms heterodimers with the GacS sensor kinase, thereby preventing the autophosphorylation of the latter kinase (7). GacS dimerized with RetS is therefore unable to activate its cognate response regulator, GacA. GacA is a positive regulator of the RsmY and RsmZ small RNAs, which sequester the global posttranscriptional regulator RsmA (8). RsmA prevents the translation of the HSI-I T6SS genes (9) and likely also affects the stability of the *tssABC1* cistron given that, in Δ*retS* planktonic cells, the expression of *tssC1* is upregulated compared to that in wild-type planktonic cultures (4). Another study also suggested that the expression of HSI-I T6SS genes is downregulated by the quorum-sensing regulators LasR and PqsR (MvfR), although this is likely an indirect regulatory effect because the binding sites for these two regulators have not been found in the HSI-I T6SS gene cluster (10). To date, a direct transcriptional regulator of *tssABC1* has not been identified.

Almost 10% of the P. aeruginosa genome is devoted to encoding transcription factors and two-component response systems (11), thus highlighting the importance of regulating gene expression at the transcriptional level in P. aeruginosa. Given the dependence of P. aeruginosa on transcriptional regulators to respond and adapt to the environment, we hypothesized that the expression of the *tssABC1* operon may, in addition to the RetS- and quorum sensing-dependent regulatory networks, be directly regulated by one or more transcription factors.

In the present study, we report the identification of PA3225, a LysR-type transcriptional regulator, which interacted with the *tssABC1* upstream regulatory region and played a small role in activating *tssABC1* expression. Interestingly, over the course of our work with the ΔPA3225 mutant, we observed that the deletion of PA3225 led to decreased susceptibility to multiple antibiotics of different classes. PA3225 was subsequently found to promote antibiotic susceptibility by additionally acting as a transcriptional repressor of the MexAB-OprM efflux pump as well as of the putative antibiotic resistance determinants encoded by PA1210, PA2864, and PA3228.

## RESULTS

### A 34-kDa protein binds the *tssABC1* promoter.

A protein pulldown approach adapted from a method described previously by Jutras et al. (12) was used to discover potential transcriptional regulators of the *tssABC1* operon. A biotinylated *tssABC1* promoter bait was incubated with colony biofilm whole-cell extracts since the expression of *tssC1* is known to be upregulated in colony biofilms (4). An unbaited bead control was used to rule out proteins that interacted with the beads themselves, while beads coupled to the promoter of *rpoD* were used to control for nonspecific DNA-binding proteins and general transcriptional machinery. Due to their different expression profiles and unrelated activities, we expected that transcription factors regulating *rpoD* would be different from those regulating *tssABC1*.

**FIG 1:**
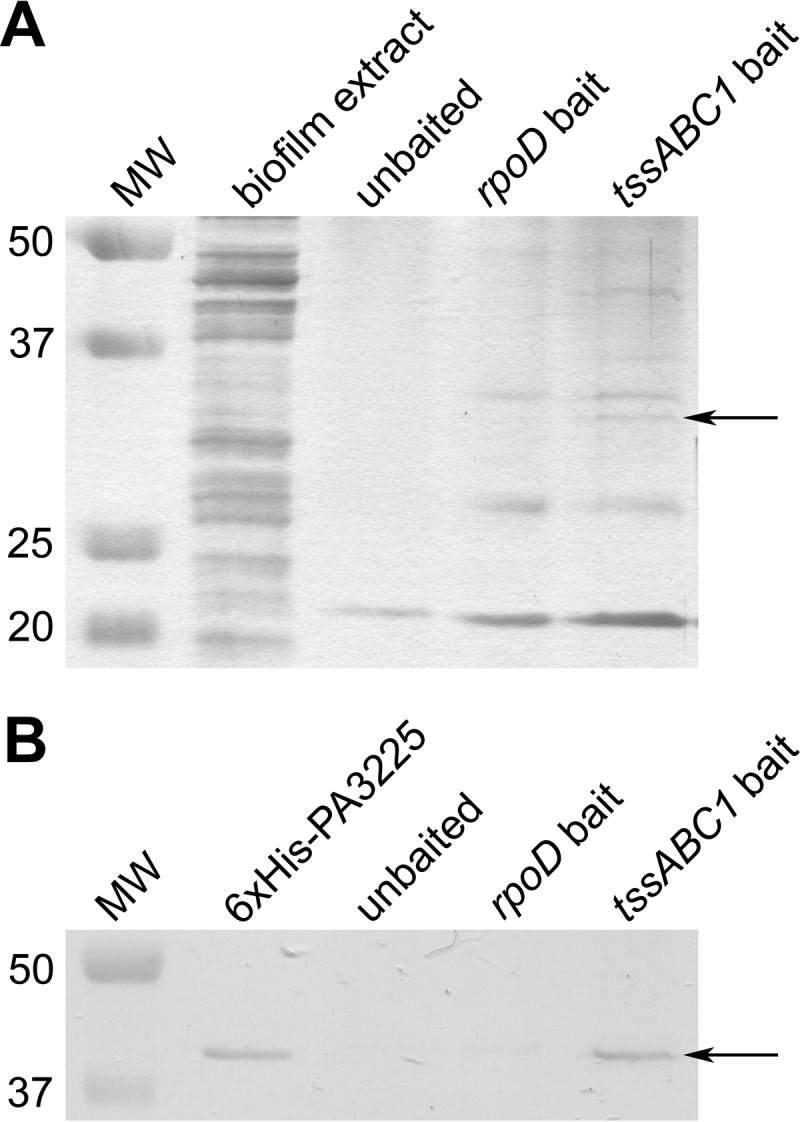
PA3225 binds to the *tssABC1* upstream regulatory region in pulldown assays. (A) The whole-cell extract from colony biofilms was incubated with streptavidin-agarose beads coupled to either no DNA or biotinylated DNA containing the upstream regulatory regions of *rpoD* or *tssABC1*. Bound proteins were visualized on a silver-stained SDS-PAGE gel, and the 34-kDa protein (shown with an arrow) that bound uniquely to the *tssABC1* DNA bait was identified as PA3225 by mass spectrometry. (B) Recombinant 6×His-tagged PA3225 was used as the sole protein input instead of the colony biofilm extract for the pulldown assay to further demonstrate that PA3225 interacts with the *tssABC1* upstream regulatory region. The protein band at 39 kDa, which corresponds to 6×His-PA3225, is shown with an arrow. MW, molecular weight (in thousands).

Proteins from the colony biofilm whole-cell extract that bound to the *tssABC1* promoter bait were separated by SDS-PAGE, and the protein bands were visualized on the gel by silver staining (Fig. 1A). Interestingly, one band at approximately 34 kDa was present in the eluate from the *tssABC1* promoter bait but not in the eluates from the *rpoD* or unbaited controls, suggesting that this protein interacted specifically with the *tssABC1* promoter bait. By using mass spectrometry, the band was shown to correspond to PA14_22470 (PA3225), a probable LysR-type transcriptional regulator that, to our knowledge, has not been previously characterized (13). Gel samples from the equivalent areas in the unbaited and *rpoD* controls were also submitted for analysis by mass spectrometry to account for background peptide hits, and no PA3225-derived peptides were identified in these controls.

### PA3225 directly interacts with the *tssABC1* promoter.

In order to confirm that PA3225 interacts specifically with the *tssABC1* promoter, recombinant PA3225 with an N-terminal 6×His tag (6×His-PA3225) was purified from an Escherichia coli BL21(DE3) strain carrying the isopropyl-β-d-thiogalactopyranoside (IPTG)-inducible pET30a-PA3225 plasmid (see Fig. S1 in the supplemental material). The pulldown experiment was repeated, this time using 2 μg of 6×His-PA3225 instead of the whole-cell colony biofilm extract as the input. As shown in Fig. 1B, recombinant PA3225 bound to the *tssABC1* promoter bait but not to the unbaited or *rpoD* negative controls.

**FIG 2:**
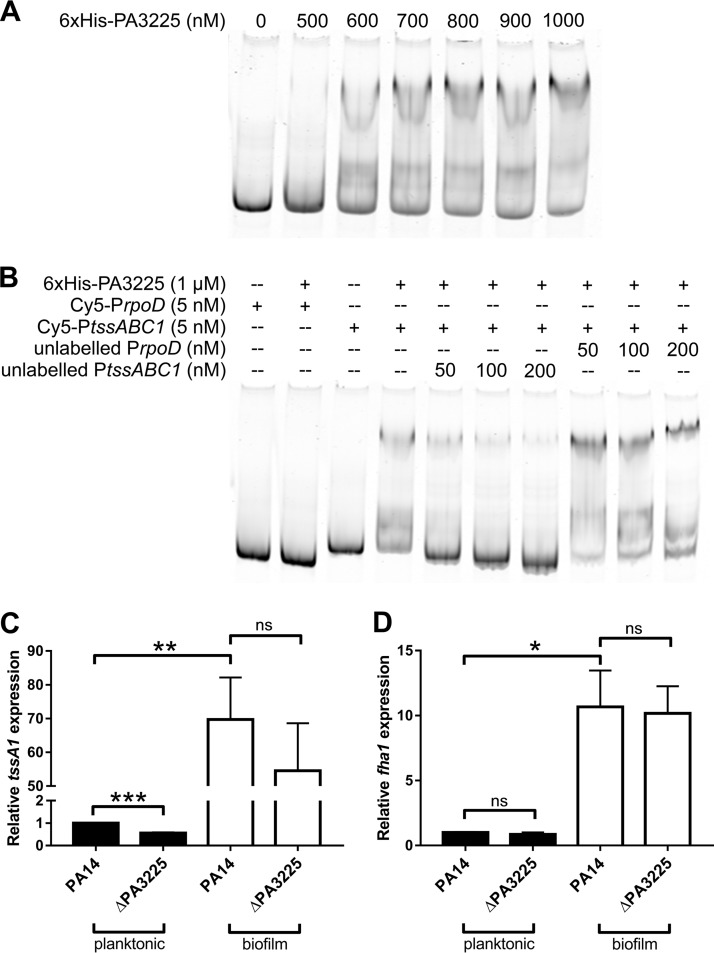
PA3225 directly binds the *tssABC1* upstream regulatory region and plays a modest role in activating *tssA1* expression. (A) EMSA showing that the *tssABC1* upstream regulatory region was bound by 6×His-PA3225. Increasing amounts of 6×His-PA3225 were incubated with 5 nM the fluorescent Cy5-P*tssABC1* probe containing the upstream regulatory region of the *tssABC1* operon, and the binding reaction mixtures were electrophoresed on a native polyacrylamide gel. (B) Competition EMSA demonstrating that 6×His-PA3225 does not bind the Cy5-P*rpoD* probe and that the unlabeled *rpoD* probe is not as effective as the unlabeled *tssABC1* probe at outcompeting the Cy5-P*tssABC1* probe for binding with 6×His-PA3225. (C) Planktonic expression of *tssA1* is decreased 2-fold in the absence of PA3225, but there is no significant decrease in *tssA1* expression in ΔPA3225 biofilms compared to wild-type biofilms as determined by qPCR. Data are shown as mean *tssA1* expression levels relative to those of wild-type planktonic cells and standard errors of the means. ***, *P* ≤ 0.001; ns, not statistically significant (as determined by two-tailed Student's *t* tests). (D) qPCR analysis demonstrates that *fha1*, which is divergently transcribed from the *tssABC1* operon, is not regulated by PA3225. However, like the *tssABC1* operon, *fha1* is also upregulated in wild-type biofilms compared to wild-type planktonic cells. Mean *fha1* expression levels relative to those of wild-type planktonic cells and standard errors of the means are shown. *, *P* ≤ 0.05; ns, not statistically significant (as determined by two-tailed Student's *t* tests).

To further demonstrate binding specificity for the *tssABC1* promoter, purified 6×His-PA3225 was used in electrophoretic mobility shift assays (EMSAs) with Cy5-labeled *tssABC1* and *rpoD* probes (Cy5-P*tssABC1* and Cy5-P*rpoD*, respectively) that were identical in sequence to the biotinylated promoter baits used in the pulldown experiment. A band with decreased electrophoretic mobility compared to that of the free Cy5-P*tssABC1* probe corresponding to the 6×His-Pa3225/Cy5-P*tssABC1* complex was observed at a 6×His-PA3225 concentration of 600 nM, and the intensity of the protein-DNA complex increased with increasing concentrations of 6×His-PA3225 (Fig. 2A). No significant shift was observed with the Cy5-P*rpoD* negative-control probe even in the presence of 1 μM 6×His-PA3225 (Fig. 2B).

In addition, a competition EMSA was performed, in which the unlabeled P*tssABC1* or P*rpoD* probe was added to binding reaction mixtures containing 5 nM the Cy5-labeled P*tssABC1* probe and 1 μM 6×His-PA3225 (Fig. 2B). When in excess, the unlabeled P*tssABC1* probe successfully outcompeted the Cy5-P*tssABC1* probe for binding to 6×His-PA3225 given that the intensity of the protein-DNA complex decreased and the amount of the free Cy5-P*tssABC1* probe increased with increasing amounts of the unlabeled P*tssABC1* probe. On the other hand, the addition of the unlabeled P*rpoD* probe did not affect the amount of free Cy5-*tssABC1* to an appreciable degree at any concentration of the P*rpoD* competitor that was tested, suggesting that the affinity of 6×His-PA3225 for the *tssABC1* promoter is much higher than that for the *rpoD* promoter. Taken together, these EMSA results suggest that 6×His-PA3225 interacts specifically with the *tssABC1* promoter region under the tested binding conditions.

### Effect of PA3225 deletion on *tssABC1* expression.

Given that our pulldown assays and EMSAs demonstrated that 6×His-PA3225 physically interacts with the *tssABC1* promoter *in vitro*, we next wanted to establish whether PA3225 has a role in regulating *tssABC1* expression *in vivo*. Consequently, an unmarked ΔPA3225 deletion mutant was constructed in the PA14 strain, and the expression level of *tssA1* in both planktonic and biofilm cultures of the PA14 and ΔPA3225 strains was determined by quantitative PCR (qPCR) (Fig. 2C). As was previously reported, the *tssABC1* transcript was upregulated in wild-type biofilms compared to wild-type planktonic cells (4). We observed an almost 2-fold decrease in the *tssA1* expression level in ΔPA3225 planktonic cells. There was also a slight decrease in the *tssA1* expression level in ΔPA3225 biofilms compared to that in wild-type biofilms; however, this difference was not statistically significant. We therefore concluded that PA3225 has a minor role in regulating *tssABC1* expression under our experimental conditions.

Previous studies have shown that the expression of the HSI-I T6SS locus is regulated posttranscriptionally by the RetS sensor kinase through a signaling cascade that converges on the posttranscriptional regulator RsmA (2). The deletion of *retS* results in a 12-fold upregulation of *tssC1* in planktonic cells (4). Since an intact RetS signaling cascade decreases *tssABC1* transcript levels, we wondered if the repressive effect of RetS on *tssABC1* expression could mask the regulatory role of PA3225. To test this hypothesis, a PA14 Δ*retS* ΔPA3225 double-deletion mutant was constructed, and the planktonic expression level of *tssA1* in this mutant was compared to that in the PA14 Δ*retS* mutant by qPCR. The expression of *tssA1* was unaffected by the deletion of PA3225 in a Δ*retS* background (data not shown).

### The T6SS gene *fha1* is transcriptionally upregulated in biofilms, but *fha1* expression does not depend on PA3225.

Since the fragment upstream of the *tssABC1* operon that was used in the pulldown experiment also corresponds to the upstream regulatory region of the HSI-I T6SS *fha1-tssJKL1* operon, the expression level of *fha1* in wild-type PA14 was compared to that in the ΔPA3225 strain by qPCR (Fig. 2D). While we showed that *fha1* was preferentially expressed in biofilms compared to planktonic cells, there was no difference in *fha1* expression levels in the ΔPA3225 strain compared to the wild type.

Overall, we have provided evidence that PA3225 binds to the *tssABC1* promoter and that this protein-DNA interaction is specific. While we were unable to demonstrate a change in the expression level of the *tssABC1* locus upon the deletion of PA3225 in this study, we feel that characterization of PA3225 is important since it is likely that PA3225 plays some role in *tssABC1* transcription that will be elucidated as more information about the regulation of the HSI-I locus becomes available.

### PA3225 is a negative autoregulator of the PA3225-PA3228 operon and is preferentially expressed in biofilms.

According to the Pseudomonas Genome Database annotation, PA3225 is a putative member of the LysR-type family of transcription regulators (13). PA3225 is predicted to form an operon with three other genes (PA3226, PA3227, and PA3228) (13), and we confirmed that these genes are cotranscribed in an operon with PA3225 by reverse transcription-PCR (RT-PCR) (see Fig. S2 in the supplemental material). Classically, a LysR-type transcriptional regulator binds directly to the promoter of the gene that encodes it, thereby repressing its own expression (14).

**FIG 3:**
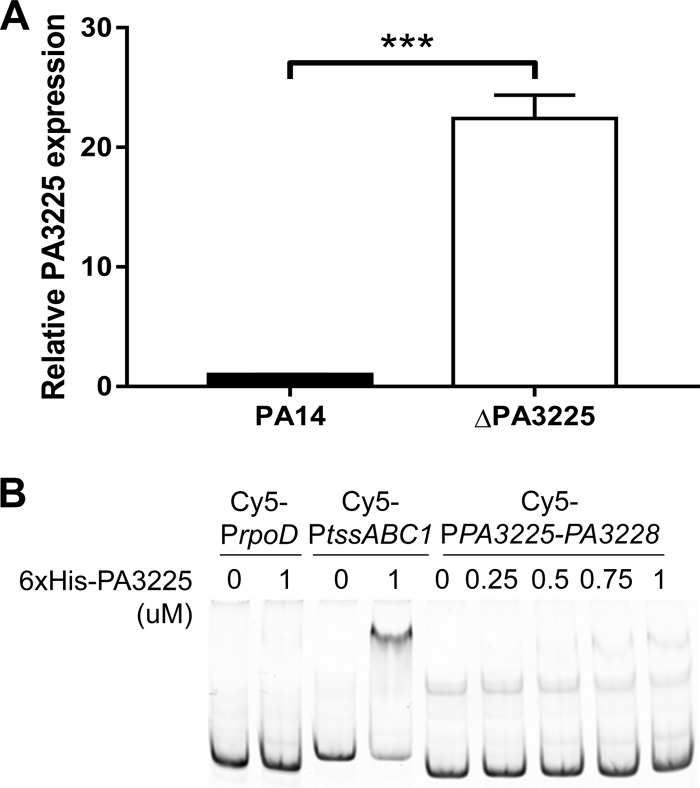
PA3225 is an autorepressor of the PA3225-PA3228 operon. (A) Deletion of PA3225 in planktonic cells results in increased expression levels of the PA3225-PA3228 locus as determined by qPCR using primers that amplify a portion of the PA3225 transcript that was not deleted during the generation of the ΔPA3225 mutant. Data are shown as mean PA3225 expression levels relative to those of wild-type planktonic cells and standard errors of the means. ***, *P* ≤ 0.001 (as determined by two-tailed Student's *t* tests). (B) PA3225 directly binds the regulatory region upstream of the PA3225-PA3228 operon. An EMSA was performed by incubating increasing amounts of recombinant 6×His-PA3225 with a Cy5-labeled DNA probe (Cy5-P*PA3225-PA3228*) corresponding to the region spanning positions −351 to +20 (relative to the translational start site of PA3225) upstream of the PA3225-PA3228 operon. Binding of 6×His-PA3225 to the Cy5-*rpoD* and Cy5-*tssABC1* probes used in Fig. 2 is included as negative and positive binding controls, respectively. Binding reaction mixtures were resolved on a native polyacrylamide gel, and the gel was visualized on a Typhoon Trio scanner.

In order to assess the regulatory effect of PA3225 on the activity of the PA3225-PA3228 promoter as well as the expression pattern of PA3225 in wild-type planktonic and biofilm cells, we performed qPCR with primers that amplified a portion of the PA3225 locus that had not been deleted in the construction of the ΔPA3225 mutant (Fig. 3A). The expression level of the PA3225 transcript in planktonic cultures increased more than 20-fold in the ΔPA3225 mutant relative to the wild type, which suggested that PA3225 is a negative autoregulator of the PA3225-PA3228 operon, as predicted. Since PA3225 was originally identified in biofilm whole-cell extracts, we next determined whether there was a difference in wild-type expression levels of PA3225 between planktonic and biofilm cells. The expression level of PA3225 was approximately five times higher in wild-type biofilms than in wild-type planktonic cells (Fig. S3). The PA3225 transcript was upregulated around 10-fold in ΔPA3225 biofilms compared to wild-type biofilms, and there was a 100-fold increase in the PA3225-PA3228 expression level in ΔPA3225 biofilms compared to wild-type planktonic cultures (Fig. S3).

To determine if PA3225 binds to its own promoter and therefore acts as a direct negative autoregulator, we performed EMSAs with recombinant 6×His-PA3225 and a fluorescently labeled DNA probe containing the sequence between positions −351 and +20 relative to the PA3225 translational start codon. The amount of shifted probe increased with increasing amounts of 6×His-PA3225 (Fig. 3B), although the affinity of 6×His-PA3225 for the PA3225-PA3228 upstream region was lower than that for *tssABC1* given that a higher concentration of 6×His-PA3225 was required to observe a visible shift in the Cy5-P*PA3225-PA3228* probe.

Overall, these qPCR and gel shift results are consistent with the conclusions that PA3225 is a transcriptional regulator that is preferentially expressed in biofilms and that PA3225 likely represses the expression of the PA3225-PA3228 operon via direct binding to the PA3225-PA3228 promoter.

### Deletion of PA3225 reduces susceptibility to multiple antibiotics in planktonic and biofilm cells.

While attempting to introduce a pUCP19 derivative (which carries a selectable marker for carbenicillin resistance) (15) into the ΔPA3225 mutant for other experiments, we noticed that the untransformed ΔPA3225 mutant was, surprisingly, able to survive when plated onto LB agar containing high concentrations of carbenicillin. Although wild-type PA14 cells were effectively killed by 300 μg/ml carbenicillin, a carbenicillin concentration as high as 1,000 μg/ml was still not effective at eliminating the ΔPA3225 mutant (D. Sparks and C. W. Hall, unpublished observations).

In order to obtain a more complete susceptibility profile of the ΔPA3225 mutant, the MICs of a panel of antibiotics of different classes were determined in both LB medium and M63 minimal medium (Table 1). For each of the two types of media, the wild-type and mutant strains shared essentially identical growth curves (data not shown). Interestingly, the ΔPA3225 mutant displayed increased planktonic resistance to several antibiotics with diverse mechanisms of action. Compared to wild-type PA14, the MIC values of the ΔPA3225 strain in LB medium were increased 2-fold for ciprofloxacin, norfloxacin, and nalidixic acid and 4-fold for carbenicillin, cefotaxime, chloramphenicol, and tetracycline. In M63 minimal medium, the MIC values were higher for the ΔPA3225 mutant than for the wild-type strain by 2-fold for tetracycline and 4-fold for ciprofloxacin, norfloxacin, nalidixic acid, carbenicillin, cefotaxime, and chloramphenicol. The MICs of gentamicin and tobramycin in both media were not affected by the absence of PA3225, suggesting that PA3225 was not important for susceptibility to these aminoglycoside antibiotics. To confirm that the phenotype of decreased multidrug susceptibility of the ΔPA3225 mutant was truly due to the absence of PA3225 and not due to polar effects of the deletion on adjacent genes, we introduced the pJB866 vector carrying the PA3225 open reading frame into the PA14 and ΔPA3225 strains. Compared to the PA14 or ΔPA3225 strain carrying the pJB866 plasmid, strains with pJB866::PA3225 generally had lower MICs for most antibiotics tested, indicating that PA3225 overexpression restored antibiotic susceptibility (Table 1).

**TABLE 1:** Impact of PA3225 deletion and overexpression on antibiotic susceptibility of P. aeruginosa in LB and M63 media

Strain	MIC in LB medium (MIC in M63 medium) (μg/ml)^*a*^								
	TOB	GEN	CIP	NOR	NAL	CAR	CTX	CHL	TET
PA14	4 (1)	8 (2)	1 (0.0625)	2 (0.5)	64 (32)	256 (128)	32 (32)	128 (16)	16 (4)
ΔPA3225	4 (1)	8 (2)	2 (0.25)	4 (2)	128 (128)	1024 (512)	128 (128)	512 (64)	64 (8)
PA14/pJB866	4 (1)	8 (2)	0.5 (0.5)	2 (2)	128 (128)	512 (128)	32 (32)	256 (16)	ND
PA14/pJB866::PA3225	4 (1)	8 (2)	0.25 (0.0625)	1 (0.25)	64 (16)	256 (64)	64 (8)	256 (16)	ND
ΔPA3225/pJB866	4 (1)	8 (2)	1 (0.25)	4 (2)	256 (128)	512 (512)	128 (32)	512 (32)	ND
ΔPA3225/pJB866::PA3225	4 (1)	8 (2)	1 (0.125)	2 (0.5)	64 (32)	512 (256)	128 (32)	256 (32)	ND

a TOB, tobramycin; GEN, gentamicin; CIP, ciprofloxacin; NOR, norfloxacin; NAL, nalidixic acid; CAR, carbenicillin; CTX, cefotaxime; CHL, chloramphenicol; TET, tetracycline; ND, not determined.

To confirm our findings that the ΔPA3225 mutant was less susceptible to various antibiotics than the wild type, we performed a drug gradient plate assay (16). In this assay, strains are streaked parallel to a linear antibiotic concentration gradient on an agar plate. Visible growth of PA14 was inhibited at lower concentrations of nalidixic acid (see Fig. S4A in the supplemental material), ciprofloxacin (Fig. S4B), norfloxacin (Fig. S4C), and tetracycline (Fig. S4D) than in the ΔPA3225 mutant, confirming that the ΔPA3225 strain was indeed less susceptible than the wild type to multiple antibiotics.

The minimal bactericidal concentration (MBC) is another metric of antibiotic susceptibility in planktonic and biofilm cells (17, 18). The MBC in planktonic cells (MBC-P) and the MBC in biofilm cells (MBC-B) of ciprofloxacin and tobramycin, two clinically important antibiotics that are used to treat P. aeruginosa infections, were determined for the wild-type and ΔPA3225 strains (Table S1). In the case of ciprofloxacin, the MBC-P for the wild-type PA14 strain was 4 μg/ml, while the MBC-P for the ΔPA3225 mutant was 8 μg/ml. Since PA3225 was preferentially expressed in biofilms, we sought to determine if the lack of PA3225 also rendered biofilms less susceptible to ciprofloxacin. The deletion of PA3225 resulted in a 2-fold decrease in biofilm susceptibility to ciprofloxacin, as the MBC-B of ciprofloxacin for wild-type PA14 was 40 μg/ml, while that for the ΔPA3225 mutant was 80 μg/ml. Consistent with the MIC results, the absence of PA3225 did not affect either the MBC-P or the MBC-B of tobramycin. It is worthwhile to note that biofilm formation of the ΔPA3225 mutant was essentially equivalent to that of the wild-type strain as determined by quantification of crystal violet staining of biofilms formed in 96-well microtiter plates (data not shown) (19).

Overall, the antibiotic susceptibility assays showed that the ΔPA3225 mutant had reduced susceptibility to multiple antibiotics when grown planktonically and that biofilms formed by the ΔPA3225 mutant had increased recalcitrance to ciprofloxacin compared to PA14 biofilms.

### PA3225 is a novel transcriptional repressor of *mexAB-oprM*.

**FIG 4:**
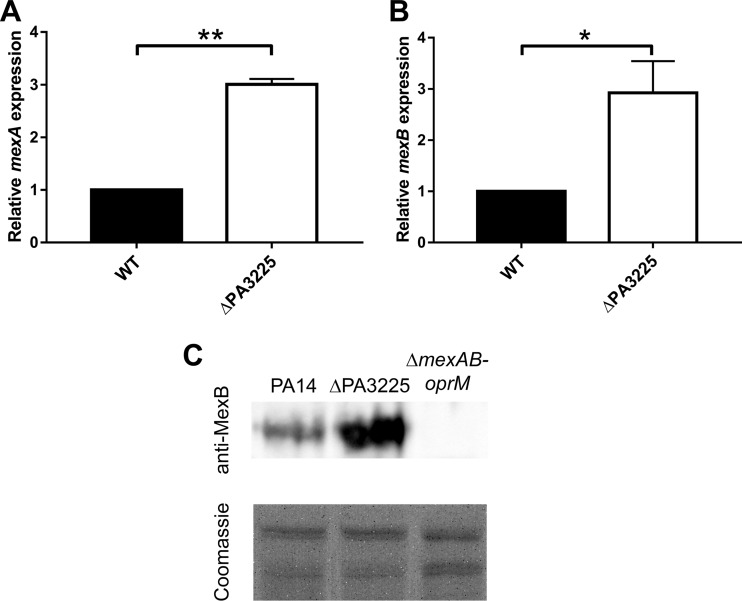
PA3225 is a novel transcriptional repressor of the MexAB-OprM multidrug efflux pump. (A and B) Expression levels of *mexA* (A) and of *mexB* (B) are approximately three times higher in planktonic ΔPA3225 cells than in planktonic wild-type cells as determined by qPCR. Data are presented as mean gene expression levels and standard errors of the means for ΔPA3225 planktonic cells relative to wild-type planktonic cells. *, *P* ≤ 0.05; **, *P* ≤ 0.01 (as determined by two-tailed Student's *t* test). (C, top) Detection of the MexB protein in cell envelopes of the PA14, ΔPA3225, and Δ*mexAB-oprM* strains by Western blotting using anti-MexB antiserum. The blot is representative of data from four experiments. (Bottom) For each Western blot, another gel was run in parallel and Coomassie blue stained to demonstrate equal loading of the gel lanes.

Given that the ΔPA3225 mutant was less susceptible to a variety of structurally and functionally unrelated antibiotics when grown planktonically, we next used qPCR to assess the expression levels of some of the P. aeruginosa RND multidrug efflux pump genes (*mexAB-oprM*, *mexCD-oprJ*, *mexEF-oprN*, and *mexXY*) in the ΔPA3225 deletion mutant (Fig. 4A and B and data not shown). We reasoned that the decreased susceptibility of the mutant could be potentially due to a loss of the PA3225-mediated transcriptional repression of multidrug efflux pumps. Interestingly, there was an almost 3-fold increase in the expression levels of *mexA* (Fig. 4A) and *mexB* (Fig. 4B) in ΔPA3225 planktonic cells compared to the wild type, suggesting that PA3225 may play a role in downregulating the expression of the MexAB-OprM efflux pump. The expression levels of the other RND multidrug efflux pumps were not affected by the deletion of PA3225 (data not shown).

Protein levels of MexB in the ΔPA3225 strain were assessed by immunoblotting to determine if the transcriptional upregulation of *mexAB-oprM* translated to an increase in MexB production. Western blotting with anti-MexB antiserum revealed an increase in the MexB abundance in the ΔPA3225 deletion mutant compared to the wild type (Fig. 4C). As expected, MexB was not detected in a Δ*mexAB-oprM* strain (Fig. 4C).

**FIG 5:**
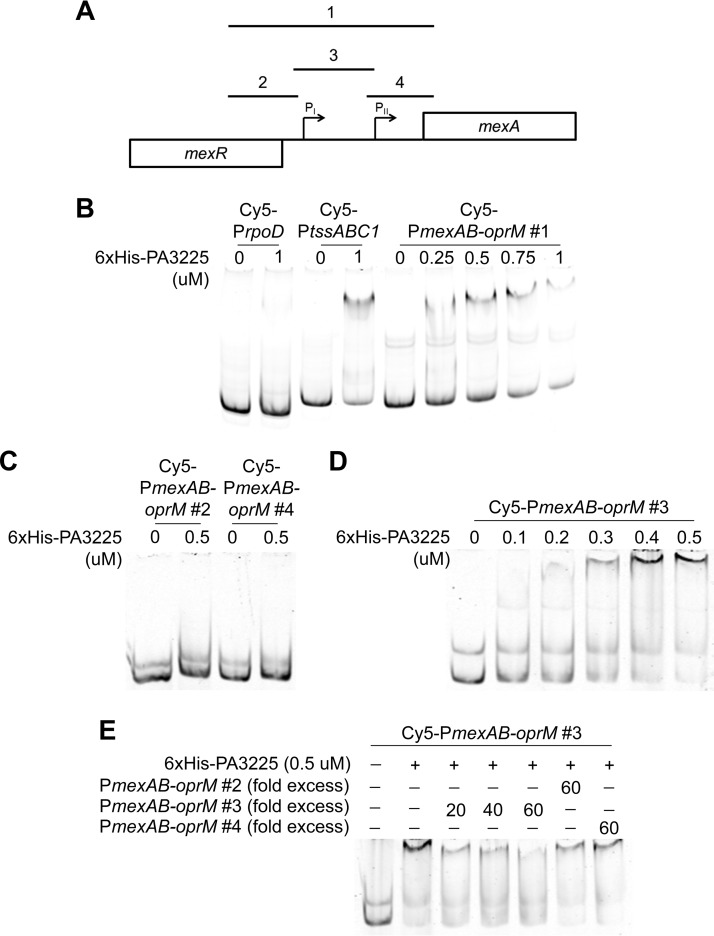
PA3225 binds to the promoter region of the *mexAB-oprM* operon. (A) Schematic showing the region upstream of *mexAB-oprM* along with the approximate locations of the EMSA probes (labeled 1, 2, 3, and 4). The locations of two previously reported *mexAB-oprM* promoters (P_I_ and P_II_) are shown with arrows (20, 33). The P_I_ promoter is located on fragment 3. (B) EMSA of Cy5-P*mexAB-oprM* probe 1 with increasing amounts of 6×His-PA3225. Binding of 6×His-PA3225 to the Cy5-*rpoD* and Cy5-*tssABC1* probes used in Fig. 2 is included as negative and positive binding controls, respectively. (C) EMSA of Cy5-P*mexAB-oprM* probes 2 and 4 demonstrating that these regions are not responsible for the observed binding of 6×His-PA3225 to the region upstream of *mexAB-oprM*. (D) EMSA showing that 6×His-PA3225 interacts with Cy5-P*mexAB-oprM* probe 3, which contains the P_I_ promoter of the *mexAB-oprM* operon. (E) The specificity of binding of 6×His-PA3225 to Cy5-P*mexAB-oprM* probe 3 was assessed by using a competition EMSA. Unlabeled P*mexAB-oprM* probe 3 (specific competitor) or unlabeled P*mexAB-oprM* probes 2 and 4 (nonspecific competitors) were incubated with reaction mixtures containing constant amounts of 6×His-PA3225 and Cy5-P*mexAB-oprM* probe 3.

To determine if PA3225 binds to the promoter of the *mexAB-oprM* operon, EMSAs were performed with the recombinant 6×His-PA3225 protein and fluorescently labeled DNA probes corresponding to various regions upstream of the *mexAB-oprM* locus (shown schematically in Fig. 5A). There was decreased mobility of Cy5-P*mexAB-oprM* probe 1 with increasing concentrations of 6×His-PA3225 (Fig. 5B), indicating that the 6×His-PA3225 protein potentially interacted with the promoter region of the *mexAB-oprM* operon. To further define where 6×His-PA3225 bound to the *mexAB-oprM* promoter region, Cy5-P*mexAB-oprM* probe 1 was subdivided into three smaller, slightly overlapping Cy5-labeled probes (Cy5-P*mexAB-oprM* probes 2, 3, and 4). While Cy5-P*mexAB-oprM* probes 2 and 4 did not bind to 6×His-PA3225 under our conditions (Fig. 5C), a shift was observed for Cy5-P*mexAB-oprM* probe 3 with increasing amounts of the recombinant 6×His-PA3225 protein (Fig. 5D). This interaction could be competed with unlabeled P*mexAB-oprM* probe 3 but not with unlabeled P*mexAB-oprM* probe 2 or 4, suggesting that the interaction between 6×His-PA3225 and P*mexAB-oprM* probe 3 is specific (Fig. 5E). Interestingly, Cy5-P*mexAB-oprM* probe 3 contains the −35 and −10 elements of the previously defined distal P_I_ promoter of the *mexAB-oprM* operon (20), suggesting that PA3225 might act at this site to repress *mexAB-oprM* expression. Future mutational studies of this promoter region will allow confirmation of this hypothesis.

To assess whether the decreased susceptibility of the ΔPA3225 strain was dependent on MexAB-OprM, we deleted the *mexAB-oprM* locus in the PA14 and ΔPA3225 backgrounds and measured the susceptibility of these strains using MIC tests (Table 2). Consistent with data from previous reports, the Δ*mexAB-oprM* strain was significantly more susceptible than wild-type strain PA14 to quinolones, β-lactams, chloramphenicol, and tetracycline. The ΔPA3225 Δ*mexAB-oprM* double-deletion mutant had essentially the same MICs as the Δ*mexAB-oprM* strain for most of the antibiotics tested. In other words, in the absence of the *mexAB-oprM* operon, the deletion of PA3225 did not reduce P. aeruginosa susceptibility, suggesting that the reduction in the antibiotic susceptibility of the ΔPA3225 mutant involves the MexAB-OprM efflux pump.

**TABLE 2:** Contribution of *mexAB-oprM* to the decreased susceptibility of the ΔPA3225 mutant

Strain	MIC (μg/ml)^*a*^								
	TOB	CIP	NOR	NAL	LVX	CAR	CTX	CHL	TET
PA14	2	0.25	0.5	64	0.25	64	16	128	16
ΔPA3225	2	0.5	2	512	1	256	64	256	64
Δ*mexAB-oprM*	4	0.03	0.125	16	0.06	1	4	32	1
ΔPA3225 Δ*mexAB-oprM*	2	0.03	0.25	16	0.06	1–2	4	32	1

a TOB, tobramycin; CIP, ciprofloxacin; NOR, norfloxacin; NAL, nalidixic acid; LVX, levofloxacin; CAR, carbenicillin; CTX, cefotaxime; CHL, chloramphenicol; TET, tetracycline.

Therefore, based upon the qPCR, immunoblot, gel shift, and drug susceptibility data, PA3225 is likely a novel transcriptional repressor of *mexAB-oprM*, and the upregulation of this efflux pump could be a factor contributing to the decreased antibiotic susceptibility of the ΔPA3225 mutant.

### The regulon of PA3225 reveals that PA3225 typically acts as a transcriptional repressor.

Since PA3225 is a predicted transcriptional regulator, the decrease in antibiotic susceptibility when PA3225 is absent suggests that PA3225 is a repressor of one or more genes whose products may be involved in antibiotic resistance. While we already demonstrated that the regulatory targets of PA3225 include the PA3225-PA3228 and *mexAB-oprM* operons, differential expression analysis of the ΔPA3225 mutant compared to the wild type was performed by using transcriptome sequencing (RNA-seq) in order to elucidate the other members of the PA3225 regulon in both planktonic and biofilm cells. Genes that were found to be significantly (log_2_-fold change of >2 or <−2) up- or downregulated in planktonic or biofilm cells of the ΔPA3225 mutant compared to planktonic or biofilm cells of wild-type strain PA14 are presented in Table 3 and Table S2 in the supplemental material, respectively. The PA3225, PA3226, PA3227, and PA3228 genes were significantly upregulated in both planktonic and biofilm cells of the ΔPA3225 strain. This finding is in agreement with our qPCR and EMSA findings that PA3225 is an autorepressor of the putative PA3225-PA3228 operon. According to the Pseudomonas Genome Database (13), PA3226 encodes a probable hydrolase, PA3227 (*ppiA*) is a peptidyl-prolyl *cis-trans* isomerase that is likely involved in the folding of periplasmic proteins (21, 22), and PA3228 is a putative ATP-binding/permease fusion ATP-binding cassette (ABC) transporter that may function as a multidrug transporter based upon the NCBI Conserved Domain Database prediction (23). Three other genes (PA1210, PA2864, and PA3229) were also significantly upregulated in planktonic and biofilm cells of the ΔPA3225 mutant compared to wild-type cells. PA1210 encodes a putative pirin protein, while PA2864 and PA3229 are hypothetical proteins with unknown functions (13). The PA1863 and PA1864 loci, which encode the ModA ABC permease involved in molybdate uptake (24) and a TetR family transcriptional regulator (13), respectively, were downregulated in ΔPA3225 planktonic cells; however, the expression levels of PA1863 and PA1864 were not significantly different between PA14 biofilms and ΔPA3225 biofilms.

**TABLE 3:** Differentially expressed genes in planktonic ΔPA3225 compared to planktonic wild-type PA14 cells

Gene	PAO1 ortholog	Predicted function^*a*^	Log_2_-fold change in expression in planktonic ΔPA3225 cells^*b*^
PA14_48650	PA1210	Hypothetical protein	+4.158
PA14_22460	PA3226	Alpha/beta hydrolase	+4.044
PA14_22450	PA3227	Peptidyl-prolyl *cis-trans* isomerase A	+3.982
PA14_22470	PA3225	LysR-type transcriptional regulator	+3.617
PA14_22440	PA3228	ABC transporter ATP-binding protein/permease	+3.418
PA14_27070	PA2864	Hypothetical protein	+3.031
PA14_22420	PA3229	Hypothetical protein	+2.314
PA14_40380	PA1864	TetR family transcriptional regulator	−4.116
PA14_40390	PA1863	Molybdate-binding periplasmic protein precursor	−2.282

a Annotated gene functions according to the Pseudomonas Genome Database (13).

b Relative to planktonic wild-type PA14 cells.

**FIG 6:**
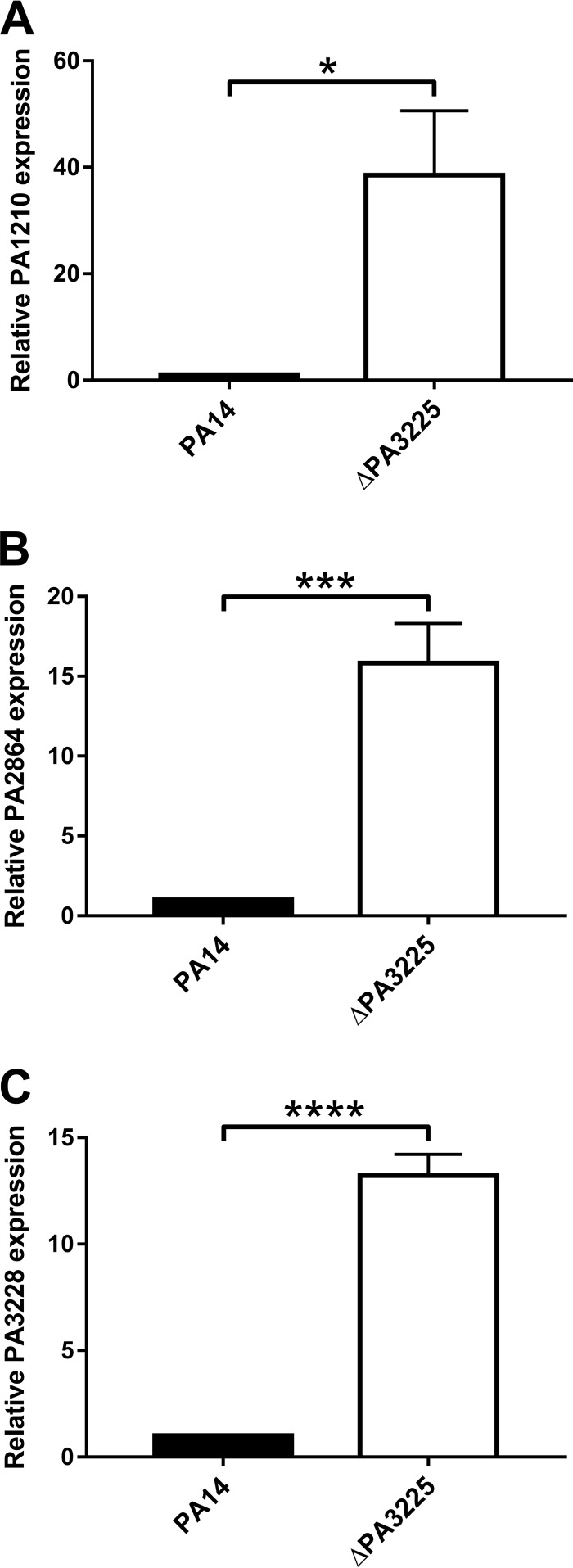
PA3225 is a transcriptional repressor of the PA1210, PA2864, and PA3228 genes. Deletion of PA3225 results in the upregulation of PA1210 (A), PA2864 (B), and PA3228 (C) in planktonic P. aeruginosa cells. The expression levels of the PA3225-regulated genes in wild-type and ΔPA3225 planktonic cultures were determined by qPCR. Mean fold changes in gene expression levels and standard errors of the means relative to the wild type are shown. *, *P* ≤ 0.05; ***, *P* ≤ 0.001; ****, *P* ≤ 0.0001 (as determined by two-tailed Student's *t* tests).

To confirm the validity of the RNA-seq data, we performed qPCR to assess the expression levels of select PA3225-regulated loci in wild-type and ΔPA3225 planktonic cultures. The expression level of PA1210 was 38 times higher in ΔPA3225 cultures than in wild-type cultures (Fig. 6A). Similarly, PA2864 and PA3228 were 15- and 13-fold more highly expressed, respectively, in ΔPA3225 planktonic cells than in wild-type cells (Fig. 6B and C). PA1210, PA2864, and PA3228 are therefore transcriptionally repressed by PA3225. While it is likely that PA3228 is directly repressed by PA3225 given that PA3228 belongs to the same operon as PA3225, we did not test whether the PA3225-mediated regulation of PA1210 and PA2864 is direct or indirect.

Since PA3228 is located in an operon with PA3225, we were concerned that the change in the expression level of PA3228 might be due to polar effects caused by the ΔPA3225 mutation. In order to address this concern, the 6×His-PA3225 allele was cloned downstream of the arabinose-inducible P_BAD_ promoter in pMQ72 (25). The resulting plasmid, pMQ72::6×His-PA3225, was transformed into the ΔPA3225 strain, and PA3228 expression was measured by qPCR. The PA3228 expression level in the ΔPA3225 strain carrying pMQ72::6×His-PA3225 was significantly lower than that in the ΔPA3225/pMQ72 strain (see Fig. S5 in the supplemental material), suggesting that the increased expression level of PA3228 in the ΔPA3225 mutant is due to a loss of PA3225 repression and not due to a polar effect from the PA3225 deletion. Moreover, this experiment confirmed that the 6×His-PA3225 protein is functional *in vivo*.

In a preliminary attempt to identify a candidate PA3225-binding site, the upstream intergenic regions of the PA3225-PA3228, *tssABC1*, *mexAB-oprM*, PA1210, and PA2864 loci were submitted to the MEME suite program (26) for *de novo* motif identification. LysR-type transcriptional regulator boxes have the general sequence TN_11_A and typically display imperfect dyadic symmetry (14). While MEME identified several motifs in common that are found in the upstream regulatory regions of these genes (data not shown), none were especially obvious or compelling candidates for a putative PA3225-binding site. We also identified the location of the transcriptional start site of PA3225 by 5′ rapid amplification of cDNA ends (RACE) (Fig. S6); however, visual inspection of the region upstream of the transcriptional start site and around a predicted PvdS sigma factor-binding site (27) also did not reveal an obviously plausible PA3225-binding site. An unbiased approach for the identification of the PA3225-binding site via chromatin immunoprecipitation sequencing (ChIP-seq) will therefore be pursued in a future study.

### PA1210, PA2864, and PA3228 are putative antibiotic resistance genes.

While we had already established that the upregulation of *mexAB-oprM* underlies, at least in part, the decreased multidrug susceptibility of the ΔPA3225 mutant, we wondered if the derepression of other members of the PA3225 regulon may also affect the antibiotic susceptibility phenotype of the ΔPA3225 strain. We therefore constructed deletion mutants of the PA1210, PA2864, PA3228, and PA3229 loci in both the wild-type and ΔPA3225 backgrounds, and we determined the MICs of several antibiotics for these mutants in LB medium (Table 4). The deletion of PA1210 and PA2864 in the wild-type PA14 background led to increased ciprofloxacin susceptibility. Additionally, the ΔPA1210, ΔPA2864, and ΔPA3228 mutants were more susceptible to levofloxacin. The ΔPA1210 and ΔPA3228 strains were also less resistant to norfloxacin and carbenicillin than the wild type. Although the deletion of PA3229 led to increased susceptibility to norfloxacin, the ΔPA3229 mutant had the same or, for some antibiotics, increased MIC values compared to those of wild-type strain PA14. MICs of strains complemented with PA1210, PA2864, and PA3228 are presented in Table 5. Complementation experiments showed that the expression of the cloned genes in the pJB866 vector could, for some antibiotics, increase the MIC.

**TABLE 4:** Contribution of PA3225-regulated genes to susceptibility of P. aeruginosa

Strain	MIC (μg/ml)^*a*^								
	TOB	CIP	LVX	NOR	NAL	CAR	CTX	CHL	TET
PA14	4	0.25	1	2	64	256	32	128	16
ΔPA1210	4	0.125	0.25	0.5	64	128	32	128	16
ΔPA2864	4	0.125	0.5	2	64	256	64	128	16
ΔPA3228	4	0.25	0.25	1	64	128	32	128	16
ΔPA3229	4	0.5	1	1	128	512	64	256	32
ΔPA3225	4	0.5	2	4	256	512	128	256	64
ΔPA1210 ΔPA3225	4	0.25	1	2	256	256	128	512	64
ΔPA2864 ΔPA3225	2	0.25	1	1	256	256	128	512	64
ΔPA3225 ΔPA3228	4	0.25	0.25	1	64	128	64	128	16
ΔPA3225 ΔPA3229	4	0.5	2	4	256	512	128	512	64

a MIC values were determined by using LB medium. TOB, tobramycin; CIP, ciprofloxacin; LVX, levofloxacin; NOR, norfloxacin; NAL, nalidixic acid; CAR, carbenicillin; CTX, cefotaxime; CHL, chloramphenicol; TET, tetracycline.

**TABLE 5:** Antibiotic susceptibilities of P. aeruginosa strains complemented with PA3225-regulated genes

Strain	MIC (μg/ml)^*a*^					
	CIP	LVX	NOR	NAL	CAR	CTX
PA14/pJB866	0.25	1	1	128	64	32
PA14/pJB866::PA1210	0.25	1	2	128	256	64
PA14/pJB866::PA2864	0.25	1	1	128	256	128
PA14/pJB866::PA3228	0.25	1	1	64	256	64
ΔPA1210/pJB866	0.25	1	1	64	128	16
ΔPA1210/pJB866::PA1210	0.25	2	1	64	256	32
ΔPA2864/pJB866	0.25	1	1	64	128	64
ΔPA2864/pJB866::PA2864	0.5	1	2	256	256	64
ΔPA3228/pJB866	0.25	0.5	0.5	64	128	32
ΔPA3228/pJB866::PA3228	0.5	1	1	128	128	64

a MIC values were determined by using LB medium. CIP, ciprofloxacin; LVX, levofloxacin; NOR, norfloxacin; NAL, nalidixic acid; CAR, carbenicillin; CTX, cefotaxime.

Since PA1210, PA2864, and PA3228 were upregulated in the ΔPA3225 strain, we hypothesized that the deletion of these genes in a ΔPA3225 background would further reveal any role that these genes might play as candidate antibiotic resistance determinants. As expected, the ΔPA1210 ΔPA3225, ΔPA2864 ΔPA3225, and ΔPA3225 ΔPA3228 double-deletion mutants were more susceptible than the parental ΔPA3225 strain to most of the antibiotics that we tested (Table 4). The upregulation of PA1210, PA2864, and PA3228 might therefore be partially responsible for the reduction in antibiotic susceptibility that we observed for the ΔPA3225 mutant, and this possibility will be explored further in future studies. PA1210, PA2864, and PA3228 also appeared to affect susceptibilities to different subsets of antibiotics. PA1210 and PA2864 were mainly involved in fluoroquinolone resistance. In contrast, PA3228 was required for resistance to a broader range of antibiotics, including β-lactams, fluoroquinolones, chloramphenicol, and tetracycline. Therefore, PA1210, PA2864, and PA3228 potentially represent novel, PA3225-regulated antibiotic resistance genes in P. aeruginosa.

## DISCUSSION

**FIG 7:**
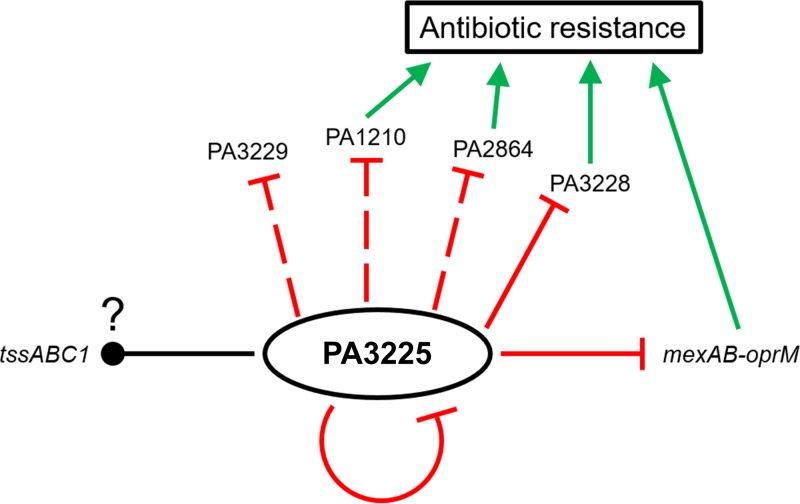
Summary schematic of the proposed mechanisms involved in the decreased susceptibility of P. aeruginosa upon deletion of the PA3225 gene. Solid red lines indicate that our EMSA results suggested that PA3225 acts as a direct transcriptional repressor of the gene, while dashed red lines indicate that we did not use an EMSA to assess whether PA3225 is a direct or indirect transcriptional repressor of the indicated locus. The solid black line with a question mark indicates that PA3225 binds directly to the promoter of *tssABC1*, but the role of PA3225 in regulating *tssABC1* expression is not entirely clear given that the *tssA1* expression level is only slightly decreased in ΔPA3225 planktonic cells and is not significantly different in ΔPA3225 biofilms compared to the wild type. Green arrows pointing toward the box indicating “Antibiotic resistance” indicate that the genes in question potentially contribute to antibiotic resistance in P. aeruginosa. Based on our data, the derepression of some or all of these genes in the ΔPA3225 mutant leads to a decrease in antibiotic susceptibility.

The major findings of this study are presented schematically in Fig. 7. This study initially sought to identify a novel transcription regulator of the *tssABC1* operon, which was previously shown to be involved in P. aeruginosa biofilm recalcitrance (4). Using a pulldown approach, we identified an uncharacterized LysR-type transcription factor, PA3225, which interacted with the promoter region of *tssABC1*. While PA3225 appeared to bind specifically to the region upstream of the *tssABC1* locus, the deletion of PA3225 resulted in only a minor decrease in the *tssA1* expression level in planktonic cells. The regulation of HSI-I T6SS expression is controlled by multiple regulatory mechanisms, such as the RetS signaling cascade (2, 4, 6) and the LasR and PqsR (MvfR) quorum-sensing systems (10). It is possible, therefore, that we did not observe a large change in the *tssA1* gene expression level in the ΔPA3225 mutant due to potential redundancy in the regulatory networks controlling the expression of the *tssABC1* operon. Further work will be required in order to better dissect the role of PA3225 in regulating *tssABC1* transcription in the context of other known regulators of the HSI-I T6SS locus.

During our characterization of PA3225, we observed that, compared to the wild-type strain, the ΔPA3225 mutant had reduced susceptibility to multiple antibiotics, including β-lactams, quinolones, chloramphenicol, and tetracycline. ΔPA3225 biofilms were additionally more resistant to ciprofloxacin than were wild-type biofilms. We found that the decreased multidrug susceptibility of the ΔPA3225 mutant could be explained, at least partially, by the transcriptional derepression of the genes encoding the MexAB-OprM multidrug efflux pump. The typical substrates extruded by the MexAB-OprM efflux pump include β-lactams, quinolones, chloramphenicol, and tetracycline but not aminoglycosides such as gentamicin (28, 29). In fact, the substrate specificities of the MexAB-OprM efflux pump mirror the susceptibility profile of the ΔPA3225 mutant. Our results from both gene and protein expression experiments strongly support a role for PA3225 in regulating the transcription of *mexAB-oprM*. Hence, PA3225 is a new addition to a group of several transcription factors that have been shown to regulate the expression of the *mexAB-oprM* locus. The transcription of the *mexAB-oprM* operon is directly repressed by MexR, and the *mexR* gene is divergently transcribed from the *mexAB-oprM* operon (20, 30–32). Interestingly, the *mexAB-oprM* operon is also directly repressed by NalD, which belongs to the TetR family of transcriptional regulators (33, 34). Additional regulators such as AmpR, BrlR, MexT (reviewed in reference 35), and CpxR (36) also affect the expression of *mexAB-oprM*. The large number of regulators that impact *mexAB-oprM* expression is a testament to the complexity of *mexAB-oprM* transcriptional regulation in P. aeruginosa. While we showed that the binding of PA3225 to the region upstream of *mexAB-oprM* and of *mexR* leads to the downregulation of *mexAB-oprM* expression, it is important to realize that we did not determine whether PA3225 represses *mexAB-oprM* directly or if PA3225 represses *mexAB-oprM* by upregulating *mexR* expression. The interplay between previously characterized *mexAB-oprM* regulators and PA3225 in regulating *mexAB-oprM* will be the topic of future investigations.

Transcriptomics analysis of the ΔPA3225 mutant via RNA-seq revealed that PA3225 also acts as a transcriptional repressor of several genes, including PA1210, PA2864, and PA3228. Unmarked deletion mutants of these three genes were more susceptible to several antibiotics, indicating that PA1210, PA2864, and PA3228 potentially encode novel determinants of low-level antibiotic resistance. Intriguingly, a mutant carrying a transposon insertion in the PA2864 open reading frame was previously shown to be less susceptible to ciprofloxacin than the wild type (37). In contrast, our susceptibility data suggested that the ΔPA2864 strain is more susceptible to ciprofloxacin than wild-type strain PA14. The reasoning behind this discrepancy is not yet clear to us.

PA3228 was previously suggested to be important for the possible development of planktonic ciprofloxacin resistance (38). Here, we demonstrated that the ΔPA3228 deletion mutants (especially in the ΔPA3225 background) were more susceptible than the parental wild-type and ΔPA3225 strains to a broad range of antibiotics, thereby suggesting a role for PA3228 in intrinsic drug resistance. PA3228 is predicted to encode a 610-amino-acid ABC transporter with both ATPase and permease domains (13, 23). Based upon our susceptibility data and bioinformatics predictions, we hypothesize that PA3228 is an ABC multidrug efflux pump. ABC transporter-type antibiotic efflux pumps are relatively rare in Gram-negative bacteria (35). However, the contribution of ABC transporters to antibiotic resistance in Gram-negative bacteria may have been underestimated. Recently, the PA4456-PA4452 operon, which encodes components of an ABC transporter and is regulated by the PhoPQ two-component system, has been implicated in P. aeruginosa resistance to tetracycline, ciprofloxacin, chloramphenicol, and trimethoprim (39). Unlike PA3228, which encodes both the ATP-binding and permease domains, PA4456 contributes the ATP-binding domain, while PA4455 possesses the permease domain (13, 39). Characterization of the putative ABC multidrug efflux pump encoded by PA3228 is under way in our laboratory.

## MATERIALS AND METHODS

### Bacterial strains, media, plasmids, and oligonucleotides.

Bacterial strains and plasmids used in this study are listed in Table 6. The wild-type P. aeruginosa strain used in this work was UCBPP-PA14 (PA14). For simplicity, all PA14 genes in this paper are referred to by the gene names of their PAO1 orthologs (11, 13). The unmarked deletion mutants were constructed by two-step allelic exchange with pEX18Gm as previously described (40, 41). Escherichia coli strains were grown in LB broth at 37°C, and P. aeruginosa strains were grown at 37°C in LB broth or M63 minimal medium supplemented with 0.4% l-arginine and 1 mM MgSO_4_. When antibiotic selection for plasmids was required, E. coli was grown in the presence of 25 μg/ml kanamycin, 20 μg/ml gentamicin, or 10 μg/ml tetracycline, and P. aeruginosa was cultured with 80 to 100 μg/ml gentamicin or 100 μg/ml tetracycline. Oligonucleotides are listed in Table 7. Most plasmids were constructed by using standard molecular cloning techniques. The pMQ72::6×His-PA3225 plasmid, which expresses the 6×His-PA3225 allele under the control of the arabinose-inducible P_BAD_ promoter, was constructed by yeast homologous gap repair exactly as previously described (25).

**TABLE 6:** Microbial strains and plasmids used in this study

Strain or plasmid	Genotype or description	Source or reference
Strains		
Escherichia coli		
DH5α	F^−^ ϕ80*lacZ*ΔM15 Δ(*lacZYA-argF*)*U169 recA1 endA1 hsdR17*(r_K_^−^ m_K_^+^) *phoA supE44* λ^−^ *thi*-*1 gyrA96 relA1*	Invitrogen
BL21(DE3)	F^−^ *ompT hsdS*_B_(r_B_^−^ m_B_^−^) *gal dcm* (DE3)	Invitrogen
S17-1	*recA pro hsdR* RP4-2-Tc::Mu-Km::Tn*7*	53
Pseudomonas aeruginosa		
TFM227	UCBPP-PA14 (wild type)	54
TFM297	PA14 ΔPA3225	This study
TFM206	PA14 Δ*retS*	4
TFM301	PA14 Δ*retS* ΔPA3225	This study
TFM434	PA14 ΔPA1210	This study
TFM431	PA14 ΔPA1210 ΔPA3225	This study
TFM436	PA14 ΔPA2864	This study
TFM433	PA14 ΔPA2864 ΔPA3225	This study
TFM59	PA14 ΔPA3228	This study
TFM409	PA14 ΔPA3225 ΔPA3228	This study
TFM435	PA14 ΔPA3229	This study
TFM432	PA14 ΔPA3225 ΔPA3229	This study
TFM504	PA14 Δ*mexAB-oprM*	This study
TFM505	PA14 ΔPA3225 Δ*mexAB-oprM*	This study
Saccharomyces cerevisiae YPH500	*MAT*α *ura3-52 lys2-801^amber^ ade2-101^ochre^ trp1*-Δ*63 his1*-Δ*200 leu2*Δ*1*	55
Plasmids		
pEX18Gm	Gene replacement vector; *sacB*; Gm^r^	40
pEX18Gm::ΔPA3225	Deletion of PA3225; Gm^r^	This study
pEX18Gm::ΔPA1210	Deletion of PA1210; Gm^r^	This study
pEX18Gm::ΔPA2864	Deletion of PA2864; Gm^r^	This study
pEX18Gm::ΔPA3228	Deletion of PA3228; Gm^r^	This study
pEX18Gm::ΔPA3229	Deletion of PA3229; Gm^r^	This study
pEX18Gm::Δ*mexAB-oprM*	Deletion of *mexAB-oprM*	This study
pET30a	His-tagged protein expression vector with IPTG-inducible T7/*lacO* promoter; Kan^r^	Novagen
pET30a-PA3225	Expression of 6×His-PA3225; Kan^r^	This study
pJB866	Expression vector with *m*-toluic acid-inducible P*_m_* promoter; Tc^r^	56
pJB866-PA3225	Overexpression of PA3225; Tc^r^	This study
pJB866-PA1210	Overexpression of PA1210; Tc^r^	This study
pJB866-PA2864	Overexpression of PA2864; Tc^r^	This study
pJB866-PA3228	Overexpression of PA3228; Tc^r^	This study
pMQ72	Yeast-Pseudomonas shuttle vector with arabinose-inducible P_BAD_ promoter; Gm^r^	25
pMQ72::6×His-PA3225	6×His-PA3225 allele from pCH02 cloned into pMQ72; Gm^r^	This study

**TABLE 7:** Oligonucleotides used in this study

Oligonucleotide	Sequence (5′–3′)^*a*^	Purpose
Pulldown experiments		
CH3	Biotin-TCAGCTTGTGGTAGCTGGTG	*tssABC1* promoter bait
CH4	AAAACGGGTACATCCAGCAC	*tssABC1* promoter bait
CH7	Biotin-CAGAGCTCGATGCAACTCATC	*rpoD* promoter bait
CH8	TGTTGCGCTTTTCCGGACAT	*rpoD* promoter bait
EMSAs		
CH16	TCAGCTTGTGGTAGCTGGTG	*tssABC1* EMSA probe
CH17	Cy5-AAAACGGGTACATCCAGCAC	*tssABC1* EMSA probe
CH20	CAGAGCTCGATGCAACTCATC	*rpoD* EMSA probe
CH21	Cy5-TGTTGCGCTTTTCCGGACAT	*rpoD* EMSA probe
CH26	AGGTTGGTGAGGAGGATGG	PA3225-PA3228 EMSA probe
CH27	Cy5-GTTACGCGTGGCGCTTTCAT	PA3225-PA3228 EMSA probe
CH29	AATCGAGCTCGCTCTGGATG	*mexAB-oprM* EMSA probe 1
CH30	Cy5-ATGGCTGGCGTTCGTTGCAT	*mexAB-oprM* EMSA probe 1
*mex*2F	Cy5-AATCGAGCTCGCTCTGGATG	*mexAB-oprM* EMSA probe 2
*mex*2R	TAGTTGACTGGATCAACCAC	*mexAB-oprM* EMSA probe 2
*mex*3F	Cy5-ATGTGGTTGATCCAGTCAAC	*mexAB-oprM* EMSA probe 3
*mex*3R	TGTAAACGTCCGAAAGCCTC	*mexAB-oprM* EMSA probe 3
*mex*4F	Cy5-GCTTTCGGACGTTTACAAAC	*mexAB-oprM* EMSA probe 4
*mex*4R	ATGGCTGGCGTTCGTTGCAT	*mexAB-oprM* EMSA probe 4
Cloning		
CH9	TTTTGGATCCATGAAAGCGCCACGCGTAAC	Cloning of PA3225 into pET30a
CH10	TTTTAAGCTTTTATCACTCGGCGGCCGGTTCG	Cloning of PA3225 into pET30a and pJB866
CH37	TAATGGTACCATGAAAGCGCCACGCGTAAC	Cloning of PA3225 into pJB866
PA3225f1	AATTGAGCTCGGTCAGGCGATTGGAACG	Deletion of PA3225
PA3225r2	AATAGGATCCATGAAGGCGGACAGCGAGC	Deletion of PA3225
PA3225f3	AATAGGATCCGAGCCGCTTCTACCTCTACA	Deletion of PA3225
PA3225r4	TAGGAAGCTTGTAGCCGCTGTCGACGTACT	Deletion of PA3225
PA3225f5	TCGCAATCGTCGATCAGCTA	Confirmation of ΔPA3225
PA3225r6	GCCAACTGGAAGCCGATCAT	Confirmation of ΔPA3225
PA1210f5	ACTAGAATTCGATGCTCGGCATGCTGCTCA	Deletion of PA1210
PA1210r6	ACATGGATCCGGCGATCTCGTCATCGTTCC	Deletion of PA1210
PA1210f7	ACATGGATCCGGCAGCATCGAGGTCAACG	Deletion of PA1210
PA1210r8	TATCAAGCTT*C*GAGGACGGCTACCTGGTGT	Deletion of PA1210
PA1210f9	CCGTCGCTGCAACGTTCCTT	Confirmation of ΔPA1210
PA1210f10	ATAACGCCGCTGCGTCGAGA	Confirmation of ΔPA1210
PA2864f3	ATTAGAATTCGATGTGCCGCTGGTGCTGGT	Deletion of PA2864
PA2864r4	ACATGGATCCGGTGACGATACGCAGGAT	Deletion of PA2864
PA2864f5	ATAAGGATCCATCACCGACAACGGCTATG	Deletion of PA2864
PA2864r6	TATCAAGCTTAGAACGCCAGCCTGTAGGAC	Deletion of PA2864
PA2864f7	GTGGCTGCGGAAGAGATAGG	Confirmation of ΔPA2864
PA2864r8	AAGCCATCCGCCAGATGCGT	Confirmation of ΔPA2864
PA3228F2	GTCTGAATTCTACACCTGGTCGGCATCAGC	Deletion of PA3228
PA3228R2	ATCAGGATCCCGAACAGCGCCACCTCGAT	Deletion of PA3228
PA3228F3	ATCAGGATCCAGAAGATCGGCCTGGTCG	Deletion of PA3228
PA3228R3	GTGTAAGCTTGTGCAGCTCGCCGCTATG	Deletion of PA3228
PA3228F1	CTACTTCCTGCGCCAGGTCT	Confirmation of ΔPA3228
PA3228R1	CGAAGCGCGTGTTAGTCGAG	Confirmation of ΔPA3228
PA3229f1	ATCTGAATTCTGTCCGGCTGGATCATGTGG	Deletion of PA3229
PA3229r2	ACTAGGATCCATGACGGCGGACACGACAGA	Deletion of PA3229
PA3229f3	ACATGGATCCCGGCGAGCTGCACAAGATCC	Deletion of PA3229
PA3229r4	TATCAAGCTTCTTGCGTCTCGCGCCAGACT	Deletion of PA3229
PA3229f5	GCTTCGTCGGCGTCGACTGA	Confirmation of ΔPA3229
PA3229r6	CCTACCTGAGCCGCGAGTTC	Confirmation of ΔPA3229
*mexA*fSacI	GCAATCGAGCTCGCTCTGGATGC	Deletion of *mexAB-oprM*
*mexB*rBamHI	ACGTGGATCCTGCGCGTGAACGAACATGC	Deletion of *mexAB-oprM*
*oprM*fBamHI	ACGTGGATCCGACCGCCTACCTGACGCTG	Deletion of *mexAB-oprM*
*oprM*rHindIII	ACGTAAGCTTCGACCTGGTGCGCATGGAT	Deletion of *mexAB-oprM*
*mexA*fDiag1	GACAACGCTGCGAAGGTCTC	Confirmation of Δ*mexAB-oprM*
*oprM*rDiag2	CAGCAGGACCAGTGCATTCT	Confirmation of Δ*mexAB-oprM*
PA1210compF	TCGGAAGCTTATGATCGAACGTCGTCCCTT	Cloning of PA1210 into pJB866
PA1210compR	ACTTGAATTCTCAGGCCACTTCCACCAGCA	Cloning of PA1210 into pJB866
PA2864compF	CCGGAAGCTTATGAATCCACTGATCAAGAC	Cloning of PA2864 into pJB866
PA2864compR	AATTGAATTCTCAGCGGGACAGCTTGCCGT	Cloning of PA2864 into pJB866
PA3228compF	TCGGAAGCTTATGCTTTATCGTCGTTTCGA	Cloning of PA3228 into pJB866
PA3228compR	AATTGAATTCTCAGTCGACGCCGACGAAGC	Cloning of PA3228 into pJB866
pMQ72::6×His-PA3225 F	ACCCGTTTTTTTGGGCTAGCGAATTCGAGCTCGGTACCCGGGGAAGGAGATATACATATGCAC	Cloning of pMQ72::6×His-PA3225
pMQ72::6×His-PA3225 R	AATCTTCTCTCATCCGCCAAAACAGCCAAGCTTGCATGCCTGCAGAAGCTTTTATCACTCGGCGG	Cloning of pMQ72::6×His-PA3225
qPCR		
*rpoD*F5	TCCTGGCCGACTACAATCGC	*rpoD* qPCR
*rpoD*R6	TTGACCGGCTCCACCTCTTC	*rpoD* qPCR
*tssA1* F1	AACCTGCTGCTGCAGAGCAA	*tssA1* qPCR
*tssA1* R1	ATACGGAAGGTGGGGTCGTT	*tssA1* qPCR
*fha1* F1	AAGGTACTGGACCAGGGACA	*fha1* qPCR
*fha1* R1	TGGTGTCGGTGAGGTAGTAC	*fha1* qPCR
PA3225f7	CGCGCATGCAGGAACAGCTC	PA3225 qPCR
PA3225r8	CCGGCCTGCTTGACCAGTTG	PA3225 qPCR
*mexA*f1	ACCTACGAGGCCGACTACCA	*mexA* qPCR
*mexA*r2	GCGTACTGCTGCTTGCTCAC	*mexA* qPCR
*mexB*f1	CCAGGTCCAGGTGCAGAACA	*mexB* qPCR
*mexB*r2	ACCACACCGACCACCATGAG	*mexB* qPCR
PA1210Qf1	CCTTCGCCGACTACTATGA	PA1210 qPCR
PA1210Qr2	CCTTCGCGGACATAGGTAAT	PA1210 qPCR
PA2864Qf1	TGGCCATCCTGCGTATCGTC	PA2864 qPCR
PA2864Qr2	CAGGCCGATGGATTCGAACC	PA2864 qPCR
PA3228Qf1	CCGAGCATGACCAACCTGAT	PA3228 qPCR
PA3228Qr2	GAGTTGCCGGTCTGCATGAT	PA3228 qPCR
5′ RACE		
PA3225 gsp1	TGGATCAGCGCTTCCTCGAC	PA3225 5′ RACE
PA3225 gsp2	CAGCCCTGTTCCATGTGGTG	PA3225 5′ RACE
RT-PCR		
PA3225-6 RT F	CGCGCATGCAGGAACAGCTC	Operon confirmation
PA3225-6 RT R	ATGCTGATGCCGACCAGGTG	Operon confirmation
PA3226-7 RT F	TCGCTGAAGCAGGCCTATTG	Operon confirmation
PA3226-7 RT R	GGTCTTCTTCTCCTGCATGC	Operon confirmation
PA3227-8 RT F	CGACAACGACTTCCTCAACC	Operon confirmation
PA3227-8 RT R	ATCAGTTCGTTGGCGTGCAC	Operon confirmation
PA3228 RT F	TGCACGCCAACGAACTGATC	Operon confirmation
PA3228 RT R	GCTTCAGCGTGGTGATGTTG	Operon confirmation

a Restriction sites are underlined.

### Protein pulldown.

The pulldown procedure was adopted from a protocol developed previously by Jutras et al. (12).

Colony biofilms were grown as previously described (42). Briefly, wild-type PA14 cultures grown overnight were spotted onto M63 minimal medium plates supplemented with 0.4% l-arginine and 1 mM MgSO_4_. Plates were incubated at 37°C for 24 h, followed by 24 h at room temperature. Biofilms were harvested and resuspended in BS/THES binding buffer (22 mM Tris-HCl [pH 7.4], 4.4 mM EDTA [pH 8.0], 8.9% [wt/vol] sucrose, 62 mM NaCl, 10 mM HEPES [pH 7.4], 5 mM CaCl_2_, 50 mM KCl, 12% glycerol) supplemented with cOmplete EDTA-free protease inhibitor cocktail (Roche) and phosphatase inhibitor cocktail 2 (Sigma-Aldrich). The cell suspension was sonicated, and the cleared lysate was recovered by centrifugation at 20,000 × *g* for 30 min at 4°C. The concentration of protein in the colony biofilm lysate was determined by using the Bradford protein assay (Bio-Rad) with bovine serum albumin (BSA) as the standard.

Biotinylated promoter baits containing the upstream regions of *tssABC1* (positions −407 to +20 relative to the *tssA1* translational start site) and *rpoD* (positions −355 to +20 relative to the *rpoD* translational start site) were amplified from PA14 genomic DNA by PCR. To perform DNA affinity chromatography, 10 μg of the *tssABC1* or *rpoD* promoter bait was incubated with 50 μl of a 4% streptavidin agarose bead slurry (Invitrogen) in coupling buffer (10 mM HEPES [pH 7.4], 100 mM NaCl, 10% glycerol, 100 μM EDTA [pH 8.0]) for 1 h at 4°C. The beads were then washed three times in BS/THES binding buffer before incubation with 600 μg colony biofilm lysate and 50 μg/ml poly(dI-dC) (Sigma-Aldrich) for 3 h at 4°C. In order to limit nonspecific binding to the beads, the beads were then washed twice with BS/THES binding buffer containing 5 μg/ml poly(dI-dC) before being resuspended in 15 μl 2× SDS-PAGE sample buffer and heated at 95°C for 5 min. Proteins bound to the promoter baits were resolved on a denaturing 10% polyacrylamide gel by SDS-PAGE, and the gel was visualized by silver staining. Protein bands were excised from the gel and were submitted to the Ottawa Institute for Systems Biology Proteomics Resource Centre for identification by high-performance chromatography–electrospray ionization tandem mass spectrometry (HPLC-ESI-MS/MS) followed by a Mascot database search (Matrix Science).

### Purification of 6×His-PA3225.

The PA3225 open reading frame was PCR amplified from PA14 genomic DNA and subsequently cloned into the BamHI/HindIII sites of pET30a by using standard methods so that the PA3225 coding sequence would have a 6×His tag at its N terminus. The pET30a-PA3225 plasmid was sequenced at the Ottawa Hospital Research Institute's StemCore Laboratories to ensure that there were no PCR-induced errors in the PA3225 open reading frame. A culture of E. coli BL21(DE3) cells carrying pET30a-PA3225 grown overnight was diluted 1:100 into LB broth, and the subculture was grown to the mid-logarithmic stage (optical density at 600 nm [OD_600_] = 0.5). IPTG was then added to the culture at a final concentration of 1 mM, and the culture was grown for an additional 3 h at 37°C. The induced cells were resuspended in binding buffer (50 mM NaH_2_PO_4_, 300 mM NaCl, 10 mM imidazole [pH 8.0]) before being lysed by sonication. The soluble lysate was incubated with Ni-nitrilotriacetic acid (NTA) agarose beads (Qiagen) in batch with rotation for 1.5 h at 4°C. The beads were then washed twice in wash buffer (50 mM NaH_2_PO_4_, 300 mM NaCl, 20 mM imidazole [pH 8.0]). 6×His-PA3225 was eluted from the beads in elution buffer (50 mM NaH_2_PO_4_, 300 mM NaCl, 250 mM imidazole [pH 8.0]). The elution buffer was exchanged for BS/THES buffer by several rounds of centrifugation in an Amicon Ultra-15 centrifugal filter unit (EMD Millipore) with a 10-kDa-molecular-mass cutoff, according to the manufacturer's instructions. The purity of 6×His-PA3225 was visually determined by SDS-PAGE (see Fig. S1 in the supplemental material), and the concentration of the purified protein was determined by using the Bradford protein assay (Bio-Rad) with BSA as the standard.

### EMSAs.

Primers labeled with a 5′ Cy5 moiety (Table 7) were used to PCR amplify fluorescent probes from PA14 genomic DNA. The P*tssABC1* and P*rpoD* probes were identical in sequence to the promoter baits used in the DNA affinity chromatography experiment. Binding reactions were performed in BS/THES binding buffer with 5 nM the Cy5-labeled probe, 10 ng/μl poly(dI-dC), and various concentrations of purified 6×His-PA3225 (ranging from 0 to 1,000 nM). For the competition EMSA, an unlabeled probe (identical in sequence to the Cy5-labeled probe but lacking the 5′ Cy5 modification) was included in the binding reaction mixture. Reaction mixtures were incubated at 4°C in the dark for 1 h. Following incubation, reaction mixtures were electrophoresed for approximately 2 h at 4°C on a nondenaturing 5% polyacrylamide gel in 0.5× Tris-borate-EDTA (TBE) buffer at 135 V. Gels were visualized by using a Typhoon Trio scanner (GE Healthcare). EMSA figures are representative of data from at least two independent experiments performed with 6×His-PA3225 that was prepared on two different occasions.

### qPCR analysis.

For RNA derived from planktonic cells, cultures grown overnight in LB medium were diluted 1:100 in M63 medium and grown for 8 h at 37°C to an OD_600_ of approximately 0.650. Colony biofilms were grown as described above. RNA was isolated from planktonic and biofilm cultures by using TRIzol reagent and the Purelink RNA minikit (Invitrogen) according to the manufacturer's recommendations, and the RNA was treated with Purelink DNase I (Invitrogen) as necessary to remove contaminating DNA. Synthesis of cDNA was performed by using 2 μg of RNA and the iScript cDNA synthesis kit (Bio-Rad). Real-time qPCR was performed on a MyiQ single-color detection system (Bio-Rad) with 20-μl reaction mixtures containing 2 μl of cDNA, 2.5 μM each gene-specific primer (Table 7), and iQ SYBR green Supermix (Bio-Rad). All qPCR experiments were performed with at least three biological replicates, each tested in triplicate. The expression level of *rpoD* was used as the internal reference standard. Fold changes were expressed as 2^−ΔΔCt^ values. Relative fold changes were graphed by using GraphPad Prism 7 software, and statistical significance was determined by using two-tailed Student's *t* tests.

### Antibiotic susceptibility assays.

MICs in LB and M63 media were determined by using the CLSI broth macrodilution method (43). Visible growth inhibition was read after incubation overnight at 37°C. MIC determinations for the PA14 and ΔPA3225 strains were performed five times.

To corroborate the MIC data, drug gradient plate assays were performed (16). Briefly, a layer of LB agar containing nalidixic acid (120 μg/ml), ciprofloxacin (0.1 μg/ml), norfloxacin (0.75 μg/ml), or tetracycline (16 μg/ml) was poured at an angle in square petri plates and allowed to solidify. The plate was placed horizontally, and the antibiotic-containing agar was then overlaid with antibiotic-free LB agar. An inoculation loop was used to streak cultures parallel to the linear antibiotic concentration gradient. Plates were incubated for 24 h at 37°C.

MBCs of tobramycin and ciprofloxacin for planktonic (MBC-P) and biofilm (MBC-B) cells were determined three times as previously described (17).

### Western blotting.

PA14, ΔPA3225, and Δ*mexAB-oprM* cell envelope proteins were isolated exactly as described previously (44). Protein concentrations were determined by using the Bradford protein assay (Bio-Rad) with BSA as the standard.

Envelope proteins (15 μg) were separated on 10% SDS-PAGE gels. For each experiment, two gels were run in parallel: one was Coomassie blue stained to visually ensure equal loading, while the other was used for Western blotting. Electrophoresed proteins were subsequently transferred onto a polyvinylidene difluoride (PVDF) membrane by using the Bio-Rad Trans-Blot Turbo Transfer system according to the manufacturer's directions (transfer parameters of 25 V, 1.3 Å, and 10 min). Membranes were blocked for 1 h at room temperature with agitation in a solution containing 1× Tris-buffered saline with 0.1% Tween 20 (TBST) plus 5% BSA. The blocked membranes were incubated overnight at 4°C with a 1:4,000 dilution of anti-MexB rabbit polyclonal antiserum (44) in 1× TBST containing 5% BSA. Following incubation with the primary antibody, membranes were washed four times in 1× TBST. Membranes were then incubated with a 1:10,000 dilution of goat anti-rabbit IgG conjugated to horseradish peroxidase (Cell Signaling Technology) in 1× TBST containing 5% BSA. After incubation with the secondary antibody, membranes were washed four times in 1× TBST and then exposed to the Clarity ECL Western blotting substrate (Bio-Rad) as recommended by the manufacturer. Imaging was performed by using an ImageQuant LAS 4010 imaging system (GE Healthcare). The Western blotting experiment was performed four times.

### 5′ RACE.

Identification of the PA3225-PA3228 transcriptional start site was performed with a 5′ RACE kit (Invitrogen) according to the manufacturer's instructions, using the gene-specific primers listed in Table 7.

### RNA-seq.

RNA was isolated from planktonic and biofilm cultures of the PA14 and ΔPA3225 strains as described above (three biological replicates under each condition for a total of 12 samples). RNA was submitted to the McGill University and Génome Québec Innovation Centre (MUGQIC) for library preparation, sequencing, and data analysis. Libraries were prepared by using the first-strand protocol, and paired-end sequencing of rRNA-depleted libraries was performed on an Illumina HiSeq 2000 instrument. Reads were trimmed by using Trimmomatic (45) and subsequently aligned to the P. aeruginosa PA14 genome (GenBank accession number CP000438.1) by using TopHat (46) and Bowtie (47) software. Raw read counts from HTSeq (48) were input into DESeq (49) and edgeR (50) for differential gene expression analysis.

### Accession number(s).

The raw and processed data from the RNA-seq experiment have been deposited in the NCBI Gene Expression Omnibus (GEO) (51, 52) under GEO series accession number GSE87213.

## Supplementary Material

Supplemental material
