# Supplementary material for: PA3225 Is a Transcriptional Repressor of Antibiotic Resistance Mechanisms in Pseudomonas aeruginosa
Source: Antimicrob Agents Chemother. 2017 Jul 25;61(8):e02114-16. doi: 10.1128/AAC.02114-16 (PMC5527654; doi:10.1128/AAC.02114-16)
Supplement: Supplemental material [file supp_61_8_e02114-16__index.html]

Supplemental material 

# PA3225 Is a Transcriptional Repressor of Antibiotic Resistance Mechanisms in Pseudomonas aeruginosa

## Supplemental material

- Supplemental file 1 -

  Fig. S1 to S6, Tables S1 and S2

  PDF, 937K
