# Supplementary material for: PA3225 Is a Transcriptional Repressor of Antibiotic Resistance Mechanisms in Pseudomonas aeruginosa
Source: Antimicrob Agents Chemother. 2017 Jul 25;61(8):e02114-16. doi: 10.1128/AAC.02114-16 (PMC5527654; doi:10.1128/AAC.02114-16)
Supplement: Supplemental material [file AAC.02114-16_zac008176411s1.pdf]

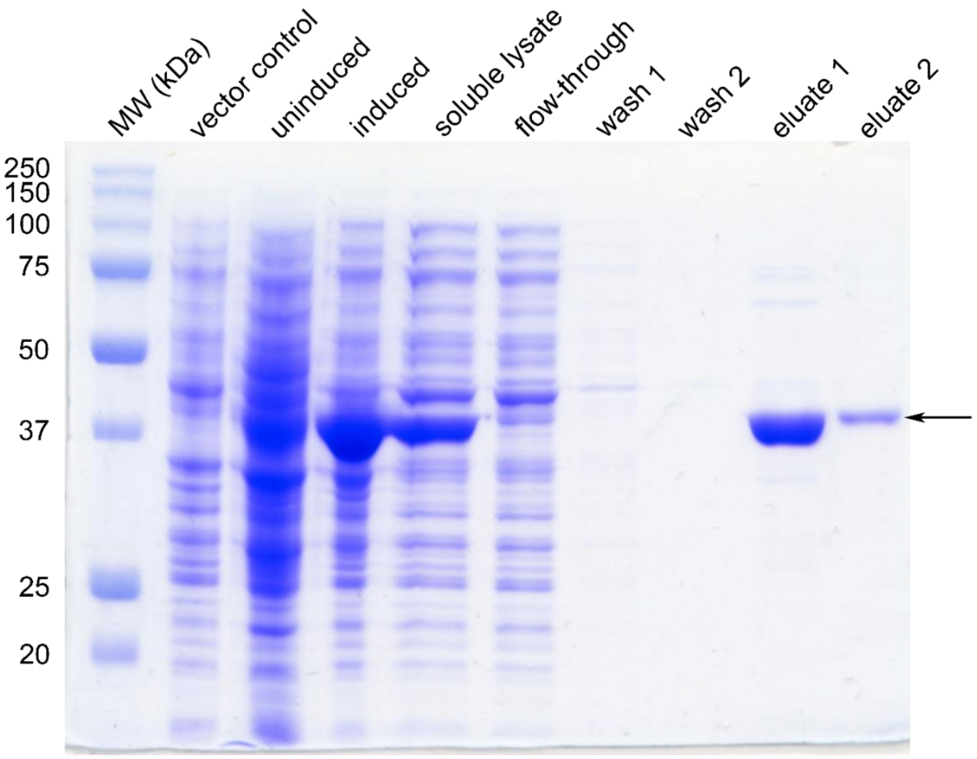


**FIG S1. Purification of 6xHis-PA3225**. Aliquots from various stages of the 6xHis-PA3225 protein purification procedure (detailed in the Materials and Methods section of the main article text) were separated by SDS-PAGE and stained with Coomassie blue. The location of 6xHis-PA3225 is shown with an arrow. "Vector control" refers to whole cell proteins isolated from IPTG-induced *E. coli* BL21(DE3) carrying pET30a. The "uninduced" and "induced" lanes contain whole cell proteins from *E. coli* BL21(DE3) pET30a-PA3225 that was either uninduced or induced with IPTG, respectively. "Soluble lysate" refers to soluble proteins following sonication of IPTG-induced *E. coli* BL21(DE3) pET30a-PA3225 cells. Soluble lysate was incubated with Ni-NTA agarose beads, and a significant portion of 6xHis-PA3225 was captured by the beads given that the flow-through had much less 6xHis-PA3225 than the input lysate. Beads were washed twice followed by two elutions with imidazole-containing buffer. Protein from eluate 2 was used for all experiments requiring 6xHis-PA3225 due to the high purity of recombinant protein in this eluate.

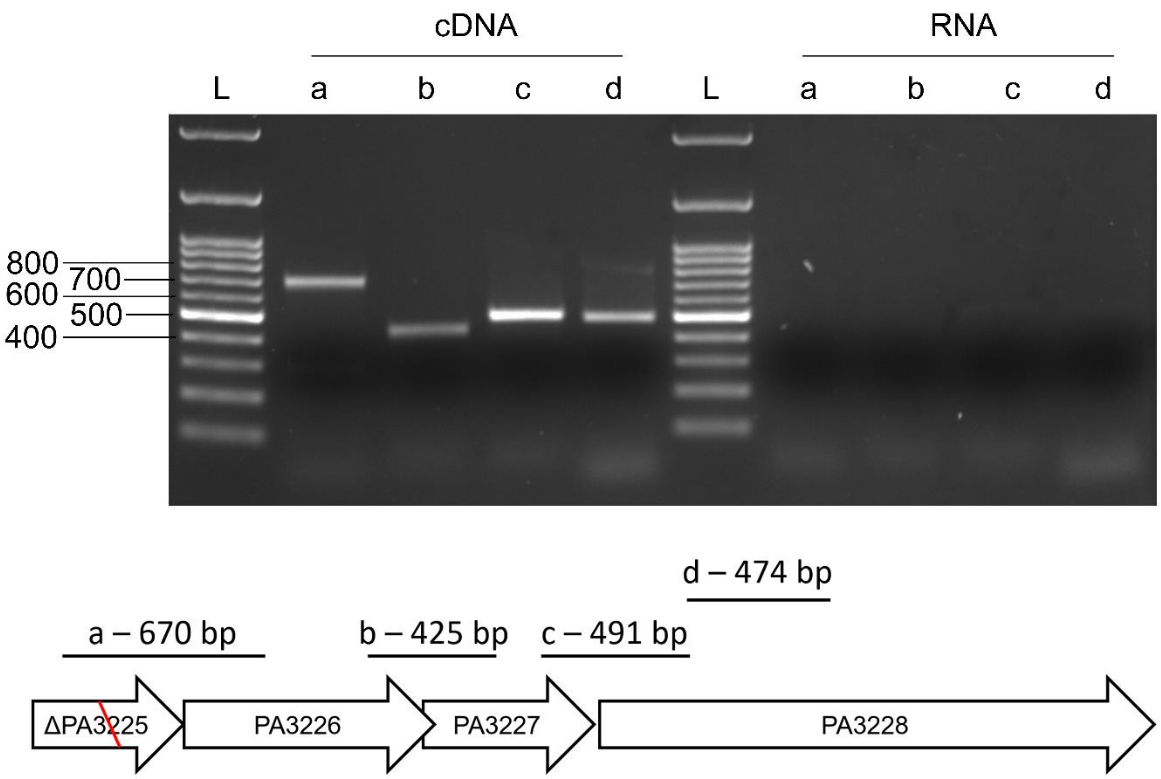


**FIG S2. The PA3225-PA3228 genes are co-transcribed.** RNA from planktonic ΔPA3225 cultures was reverse transcribed to cDNA, which was subsequently used as a template for PCR with primer sets (see **Table 2**) that amplified various regions of the predicted PA3225-PA3228 operon (regions "a" to "d" shown schematically in lower panel). PCR reactions performed with RNA in the absence of reverse transcriptase were included as controls to demonstrate the absence of contaminating genomic DNA.

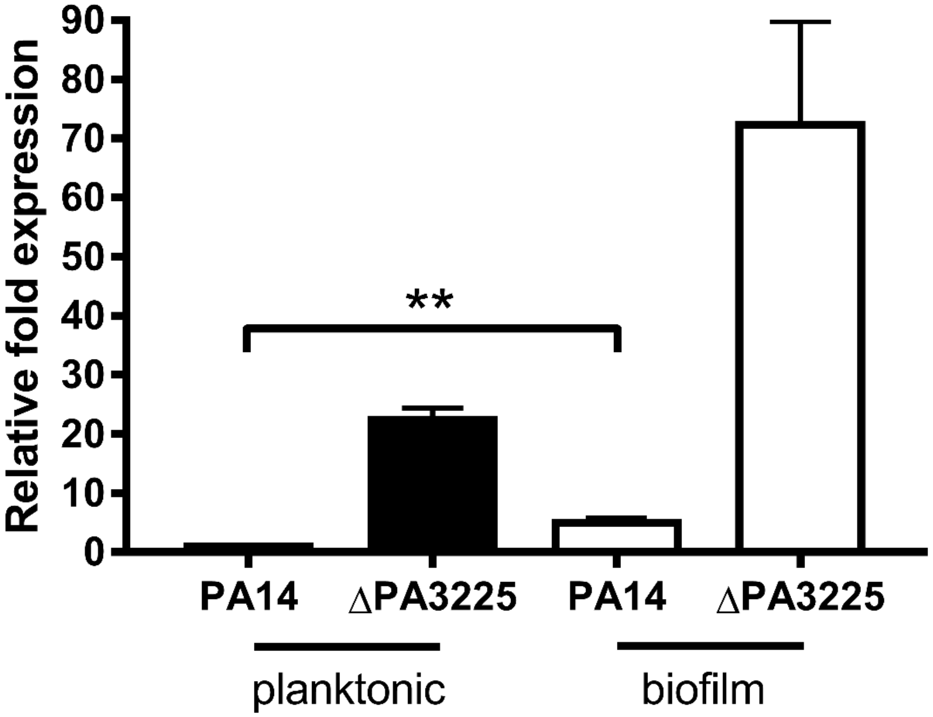


**FIG S3. PA3225 is upregulated in biofilms.** Deletion of PA3225 resulted in increased expression of the PA3225-PA3228 locus as determined by qPCR using primers that amplified a portion of the PA3225 transcript that was not deleted in the generation of the ΔPA3225 mutant. Additionally, in wild-type PA14, PA3225 expression in biofilms was 5-fold higher than that in planktonic cells. Data are shown as mean PA3225 expression relative to wild-type planktonic cells + SEM. ** $p \leq 0.01$ as determined by two-tailed Student's *t*-tests.

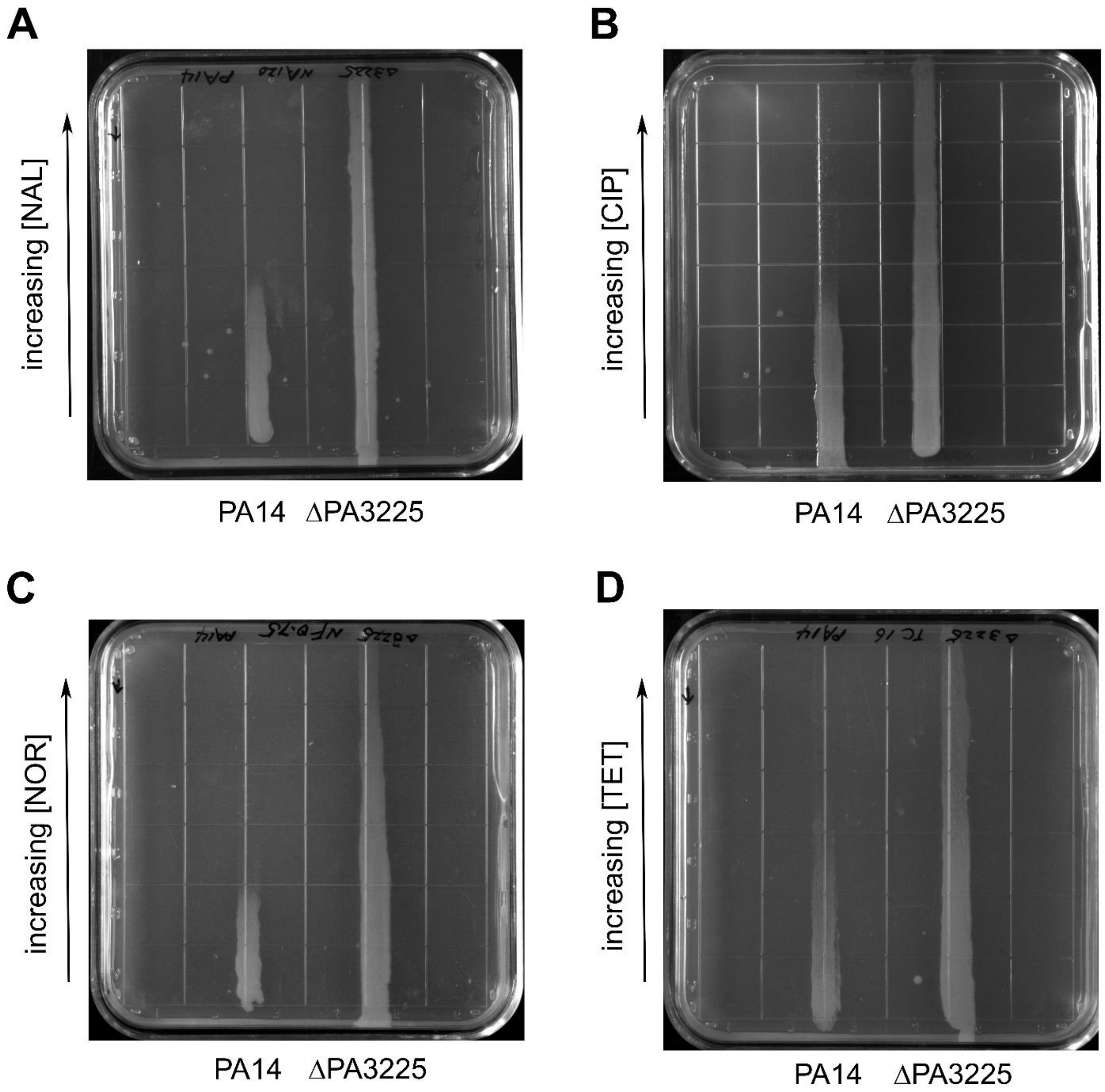


**FIG S4. Drug gradient plate assays demonstrate that the ΔPA3225 mutant has decreased antibiotic susceptibility compared to wild-type.** PA14 and ΔPA3225 were struck on LB agar plates containing a concentration gradient of (**A)** nalidixic acid (0 to 120 μg/mL), (**B)** ciprofloxacin (0 to 0.1 μg/mL), (**C)** norfloxacin (0 to 0.75 μg/mL), and (**D)** tetracycline (0 to 16 μg/mL). In all cases, the ΔPA3225 mutant had visible growth at a higher antibiotic concentration than the wild-type PA14 strain.

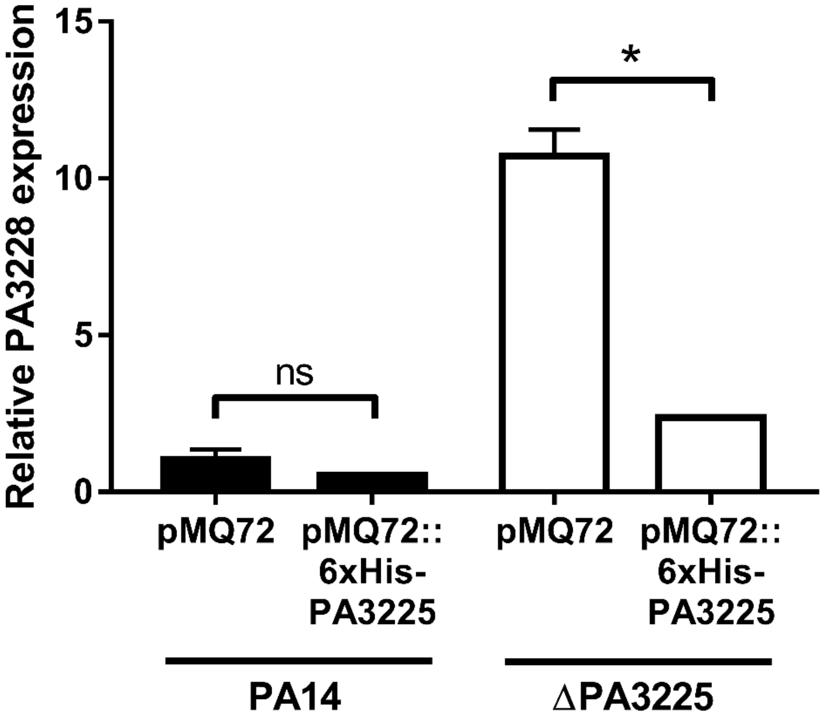


**FIG S5. Overexpression of 6xHis-PA3225 reduces PA3228 expression.** PA14 and ΔPA3225 strains carrying pMQ72 and pMQ72::6xHis-PA3225 were grown to mid-exponential phase and then induced for two hours with 1% (w/v) arabinose. Expression of PA3228 was measured by qPCR relative to PA14 pMQ72. Shown is the mean relative expression + SEM from two biological replicates, each tested in triplicate. * $p \leq 0.05$, ns = not statistically significant as determined by two-tailed Students *t*-tests

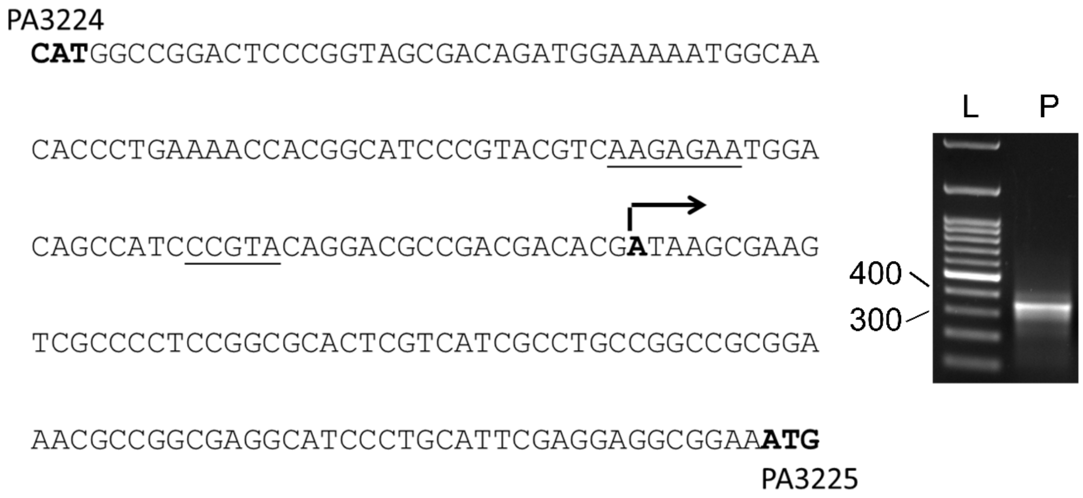


**FIG S6. The PA3224-PA3225 intergenic region**. 5’ RACE was used to identify the transcriptional start site of PA3225 (shown bolded with a bent arrow in the sequence on the left). A putative bipartite PvdS sigma factor binding site is underlined in the sequence. Translational start codons of PA3224 and PA3225 are bolded. In the right panel, the 5’ RACE product is shown in lane P and a size marker is shown in lane L.

**TABLE S1. Minimum bactericidal concentrations for planktonic (MBC-P) and biofilm (MBC-B) wild-type and ΔPA3225 cells.**

| | Tobramycin (μg/ml) | | Ciprofloxacin (μg/ml) | |
|---|---|---|---|---|
| **Strain** | **MBC-P** | **MBC-B** | **MBC-P** | **MBC-B** |
| **PA14** | 32 | 100 | 4 | 40 |
| **ΔPA3225** | 32 | 100 | 8 | 80 |

**TABLE S2. Differentially expressed genes in ΔPA3225 compared to wild-type PA14.**

| Gene name | PAO1 ortholog | Predicted function[a] | $\log_2$ fold change in planktonic ΔPA3225[b] | $\log_2$ fold change in ΔPA3225 biofilms[c] |
|---|---|---|---|---|
| PA14_48650 | PA1210 | Hypothetical protein | + 4.158 | + 2.964 |
| PA14_22460 | PA3226 | Alpha/beta hydrolase | + 4.044 | + 4.345 |
| PA14_22450 | PA3227 | Peptidyl-prolyl cis-trans isomerase A | + 3.982 | + 4.151 |
| PA14_22470 | PA3225 | LysR-type transcriptional regulator | +3.617 | + 3.929 |
| PA14_22440 | PA3228 | ABC transporter ATP-binding protein/permease | + 3.418 | + 3.773 |
| PA14_27070 | PA2864 | Hypothetical protein | + 3.031 | + 3.263 |
| PA14_22420 | PA3229 | Hypothetical protein | + 2.314 | + 2.388 |
| PA14_40380 | PA1864 | TetR family transcriptional regulator | - 4.116 | No significant difference |
| PA14_40390 | PA1863 | Molybdate-binding periplasmic protein precursor | - 2.282 | No significant difference |

[a] Annotated gene functions as per the *Pseudomonas* Genome Database (12)

[b] Relative to planktonic wild-type PA14

[c] Relative to wild-type PA14 biofilms
